# MolE8: finding DFT potential energy surface minima values from force-field optimised organic molecules with new machine learning representations†

**DOI:** 10.1039/d1sc06324c

**Published:** 2022-05-28

**Authors:** Sanha Lee, Kristaps Ermanis, Jonathan M. Goodman

**Affiliations:** Yusuf Hamied Department of Chemistry, University of Cambridge Lensfield Road Cambridge CB2 1EW UK jmg11@cam.ac.uk; School of Chemistry University Park Nottingham NG7 2RD UK Kristaps.Ermanis@nottingham.ac.uk

## Abstract

The use of machine learning techniques in computational chemistry has gained significant momentum since large molecular databases are now readily available. Predictions of molecular properties using machine learning have advantages over the traditional quantum mechanics calculations because they can be cheaper computationally without losing the accuracy. We present a new extrapolatable and explainable molecular representation based on bonds, angles and dihedrals that can be used to train machine learning models. The trained models can accurately predict the electronic energy and the free energy of small organic molecules with atom types C, H N and O, with a mean absolute error of 1.2 kcal mol^−1^. The models can be extrapolated to larger organic molecules with an average error of less than 3.7 kcal mol^−1^ for 10 or fewer heavy atoms, which represent a chemical space two orders of magnitude larger. The rapid energy predictions of multiple molecules, up to 7 times faster than previous ML models of similar accuracy, has been achieved by sampling geometries around the potential energy surface minima. Therefore, the input geometries do not have to be located precisely on the minima and we show that accurate density functional theory energy predictions can be made from force-field optimised geometries with a mean absolute error 2.5 kcal mol^−1^.

## Abstract 2

New representations and machine learning calculate DFT minima from force field geometries.

## Graphical abstract

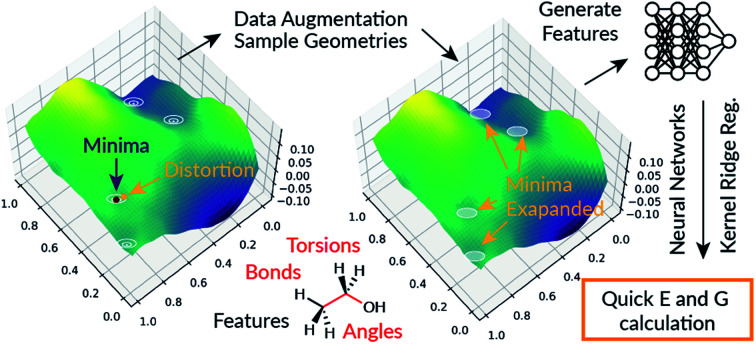

## 1 Introduction

Computationally modelling the chemical properties of molecules is essential in many areas of chemistry, molecular biology and drug design. One of the most popular quantum mechanical methods in theoretical chemistry is density functional theory (DFT).^1,2^ Although the DFT method has more accessible computational cost than the higher-precision coupled-cluster theory,^3^ the approach is still too expensive for calculating a large number of molecules. A major research focus in recent years is developing efficient computational approaches to explore chemical space.^4^ This requires a very large number of energy calculations and the speed of DFT methods is a limiting factor.

Machine learning (ML) approaches to model the properties of molecular systems statistically have gained much popularity in many areas of chemistry.^5,6^ ML methods have already been applied to improve the accuracy of the quantum mechanical methods,^7–10^ to speed up the calculations,^11–13^ to design new materials^14,15^ and to generate force-field parameters^16–18^ for molecular dynamics simulations. One of the most popular data-driven approaches is designing approximations that can replicate *ab initio* results without compromising accuracy.^19^ Currently available computationally-inexpensive practices such as the semi-empirical^20,21^ methods or classical force-field methods are efficient but lack the accuracy to model chemical reactions or molecular properties.^18^ Furthermore, unlike the electronic and free energies from DFT methods, force-field energies are not comparable for different systems because they are usually parameterised to study a particular phenomenon. Therefore, some chemical properties, such as estimating the thermodynamic selectivity of different reactions, are not possible.

The ML potential on the other hand can predict molecular energies at a fraction of the computational cost of DFT approaches with an accuracy superior to the classical force-field and semi-empirical methods.^19^ The progress of the ML potentials relies on the availability of high quality data. Reymond *et al.* explored the chemical compound space of organic molecules and generated data sets covering billions of 2D structures and SMILES strings.^22–25^ Since then, the GDB database has been exploited to develop many ML models. Roitberg *et al.* calculated over twenty million off-equilibrium conformations of organic molecules from the GDB dataset.^26^ The ANI-1 ML potential developed from this database can predict atomisation energies of organic molecules consisting of atom types H, C, N and O, up to 8 non-hydrogen atoms.^27,28^ The ANI potential has been extended to cover sulfur and halogens^29^ and coupled-cluster molecular properties.^30,31^ Lilienfeld *et al.* developed ML potentials from the GDB database to predict atomisation energies using a variety of different representations, including Coulomb matrices, bag of bonds, atomic radial distribution function based features and histogram of distances and angles.^19,32–34^ Parkhill *et al.* developed a bond-centered neural network to predict energies of molecules.^35,36^ Barros *et al.* developed Hierarchically Interacting Particle Neural Network (HIP-NN) to model molecular properties.^37^ Paton *et al.* calculated over 200 000 organic radical species containing C, H N and O atoms.^38^

The accuracy of ML potentials are highly dependent upon the choice of molecular representation used to train the model.^39^ Behler introduced several features the representations must follow in order for them to be useful in ML models.^40,41^ The representation must be rotationally and translationally invariant, the results should be invariant upon permutation of atoms of the same element and the molecule's conformation must be described in a unique way given a set of atomic positions and types. Many representations have since been developed.^27,32–37,39,42–44^ However, the representations in the literature are either not extrapolatable to molecules outside the training set or only have been tested on a small set of ‘out-of-the-box’ molecules. Furthermore, many representations require a substantial number of data points (well beyond 1 million molecules) to train and therefore are computationally expensive. Moreover, the published representations are also not ‘explainable’ in regard to what molecular features contribute to the molecular energy or ignores contributions from features such as angles and dihedrals completely.

Herein, we present a new molecular representation which is explainable for molecular property predictions. Using our feature set, we achieve state-of-the-art data efficiency in our test set molecules and in larger molecules, featuring complex ring systems, non-covalent intramolecular interactions and other challenging features. In combination, our representation is able to effectively extrapolate from 57 thousand training molecules to a five million molecule chemical space with remarkable accuracy at a very small computational cost.

## 2 Computational methods

### 2.1 Database generation

The database is based on the previously reported GDB13, GDB17 (ref. 22–24) and QM9 datasets.^19^ All molecules with 8 or fewer heavy atoms were imported from QM9. Any molecules with eight or fewer atoms in GDB13 and GDB17 but missing from QM9 were then added to our database. For the newly added molecules, 3D geometries were first generated using RDKit, and then optimized using Gaussian 16 at B3LYP/6-31G(2df,p) level of theory. Geometries imported from QM9 were resubmitted for geometry optimisation to both verify them and make data extraction consistent for all molecules in the dataset. For all converged geometries frequency calculations were also performed. This expanded the raw number of 8 heavy atom molecules from less than 22k in QM9 to more than 59k in our dataset.

Generated datasets often contain errors. Therefore, database cleaning is essential part of machine learning studies.^45^ 59 097 molecules were successfully optimised with Gaussian 16 (ref. 46) without fragmentation. We then removed the molecules with imaginary frequencies (688 molecules), fluorines (44 molecules), any bonds longer than 1.6 Å (313 molecules) and tetravalent N atoms (charged N atoms, 11 molecules). We removed molecules with four connectivity N atoms because we wanted to exclude zwitterions from the database. The molecules with long bonds are unusual and highly strained. They are too uncommon for the algorithms to successfully learn and some example structures are listed in ESI, Section 1.3.† These interesting structures will be added back in the future when additional training data are available. We further removed 75 molecules that have a 3-connectivity carbon and all three neighbouring atoms are either hydrogens with 1 connectivity, oxygens with 2 connectivity or nitrogens with 3 connectivity. These molecules have carbon atoms that do not complete the octet and few examples structures that are removed are listed in ESI, Section 1.1.† The final category for exclusion is molecules with tetravalent carbon with large bond angles, ESI, Section 1.2.† Overall, around 1900 molecules were removed from the database, and 57 143 molecules remain.

### 2.2 Molecular representations

**Fig. 1:**
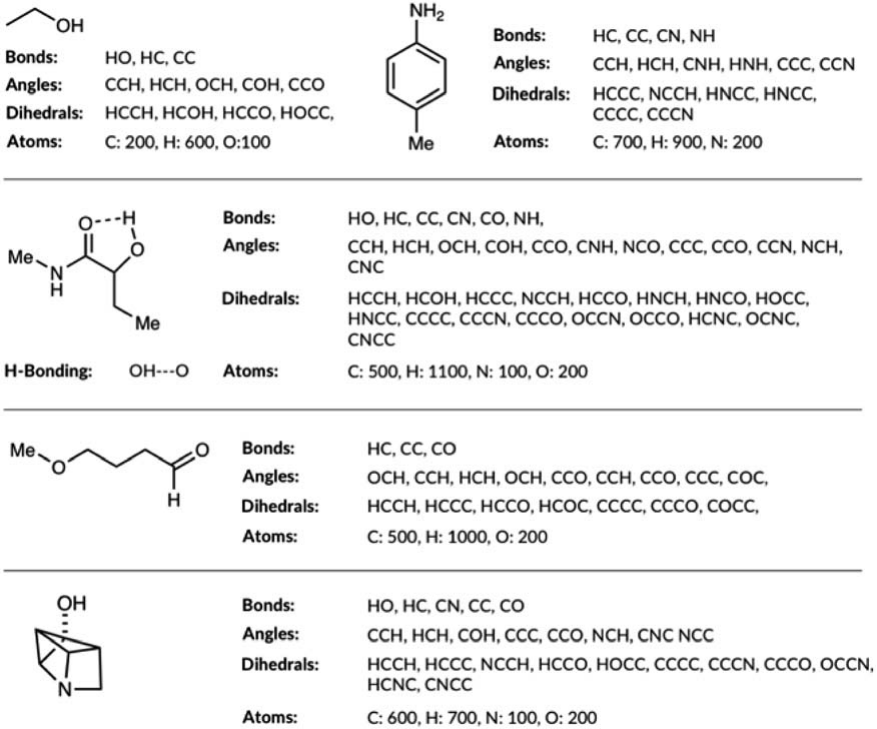
List of bonds, angles, dihedrals and atom types the feature generation algorithm has detected in few example organic molecules.

The molecular representations have been generated from the distributions of molecular features produced from the generated GDB8 database. Specifically, the representation has six contributing features: the atom types, the bond types, the angle types, the dihedral types, the hydrogen bonding (H-bonding) types and the number of NH_*x*_ groups. We list the bonds, the angles, the dihedrals and the atom types found in the four example molecules from the database in Fig. 1. The atom types are simply the number of C, H, N and O atoms, weighted by 100. The number of NH_*x*_ groups is the number of secondary (NH) or primary (NH_2_) amine *etc.* found in the molecule.

**Fig. 2:**
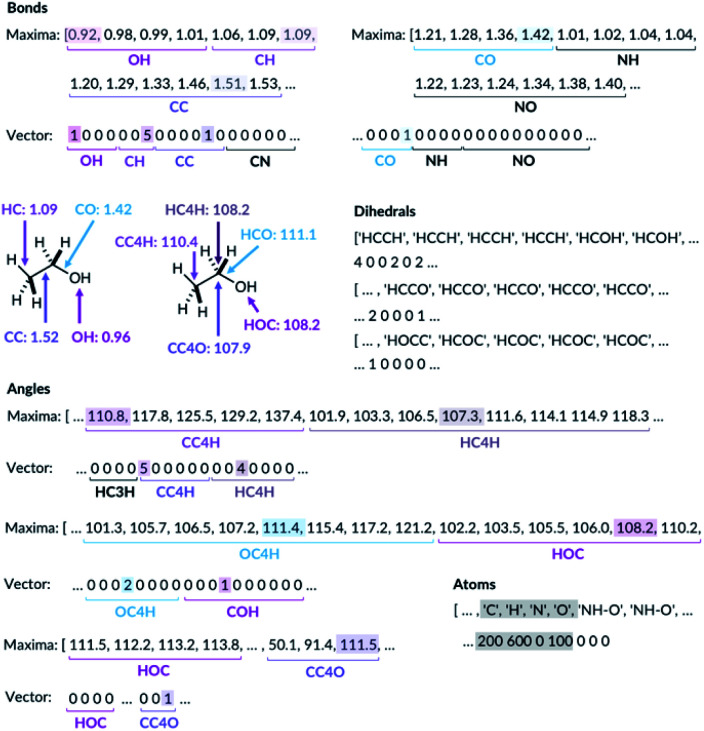
Ethanol molecule representation example. C3 and C4 are carbon atoms with 3 and 4 connectivity, respectively.

For the bond types, the feature generation algorithm loops through every single molecule in the database and extracts all the bond lengths. All the bonds in the molecule have been identified with Open Babel.^47^ The lengths are then categorised, depending on the atoms forming the bond. For example, the carbon–carbon bond lengths would be classified to the CC bond type whereas the carbon–hydrogen bond would be classified to the CH bond type. Histogram plots are then generated for each bond types found in the database and the kernel density estimation (KDE) from the SciPy library^48^ is then performed. Each maximum in the KDE plot becomes a feature. All KDE maxima are flanked by two minima, one on the longer and one on the shorter bond length side. The minima are used to assign each bond to a particular feature. The representation is then created for molecules by counting the number of times a value matching each maximum is observed in the molecule, Fig. 2. For example, the ethanol molecule has one HO bond, five HC bonds and one CC bond. Therefore, the KDE maxima features corresponding to the HO bond of length 0.96 Å and the CC bond of length 1.52 Å found in ethanol are set to 1 each and the feature corresponding to the HC bonds of length around 1.09 Å is set to 5, Fig. 2. All the other bond features in the feature vector are set to 0. The algorithm ensures that the bond types are looped through in the same order such that the features are always concatenated consistently.

The feature generation algorithm works similarly for the angle and the dihedral types. The angles and the dihedrals are also categorised, depending on the atoms forming the angle or the dihedral. The KDE plot maxima for each angle and dihedral types become the features and the representation is generated by counting the number of times each maximum is observed for each molecule. For example, the ethanol molecule has five CCH, four HCH, two OCH, one COH and one CCO angles, Fig. 2. Therefore, the features corresponding to these angle values are set to 5, 4, 2, 1 and 1, respectively. The same procedure is then repeated for all the dihedrals found in the ethanol molecule.

The molecules with OH or NH bonds can potentially form intramolecular hydrogen bonds. Since the database only contains C, H, N and O atom molecules, there are four possible H-bonding interactions: OH–O, OH–N, NH–O and NH–N. The feature generation algorithm identifies all the H-bond donor hydrogens (hydrogens in the molecule bonded to N or O) and finds all the potential H-bond acceptors (N or O atoms). If the donor–acceptor distance is between 1.3 and 2.6, then this is considered to be the H-bonding interaction.^49^ The algorithm collects all the H-bonding interactions in the database and categorise them to the four possible H-bonding groups. Histogram plots are then generated and the KDE maxima again becomes H-bonding features, similar to the bond feature generation methods. The H-bonding representation is then created for all the molecules by counting the number of times each H-bond maximum is observed in the molecule. In the ethanol example, no intramolecular H-bonding is present, and so all the H-bonding features are set to zero.

**Fig. 3:**
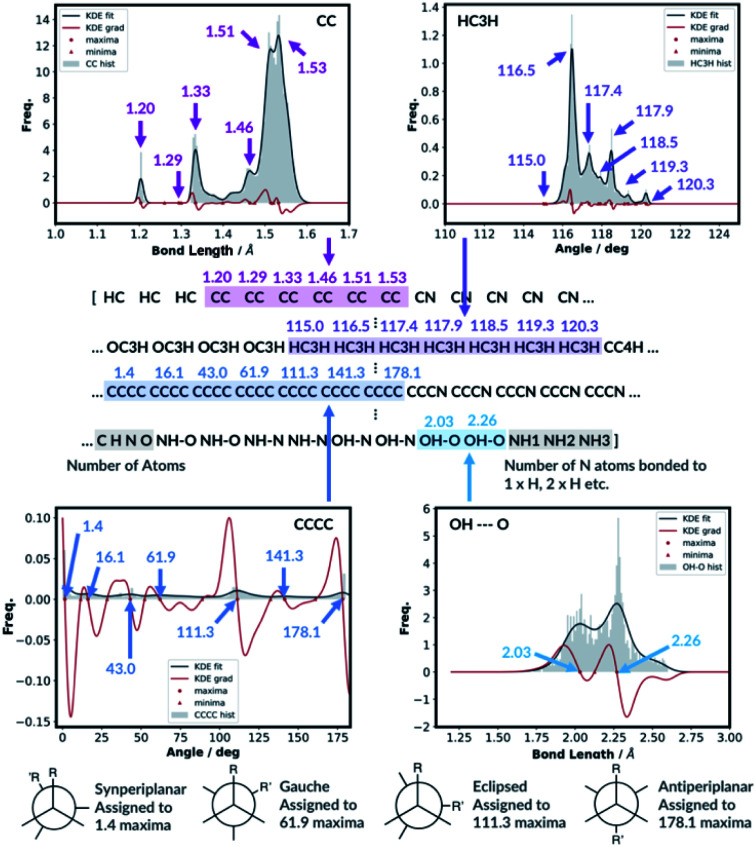
KDE plots for CC bond, HCH angle, CCCC dihedral and OH⋯O H-bonding distributions. Every maximum in the KDE plot becomes a feature in the molecular representation.

Whilst we engineer the method to think in terms of bonds, angles and dihedrals, we do not hand-engineer the specific features. The choice of the features is data driven (Fig. 3).

## 3 Results and discussion

### 3.1 Trial A: selecting the optimal representation

Once the dataset was generated and cleaned, we explored the distributions of the organic molecules within the GDB8 database. Understanding the distribution of the dataset is important because the training and the test set must be representative of the intended usage. We decided to group the molecules by stoichiometry because the number of each atom type the molecule has is the single largest contributor to the molecular electronic energy. 407 unique stoichiometries were found in the present dataset, ESI Section 2.1.† The number of molecules in each stoichiometry varies from 1 (*e.g.* N_5_H) to 2491 (C_6_NOH_11_). We investigated the distributions of molecular energies within each stoichiometry. In the majority of the cases, the maximum minus the minimum energy ranges do not overlap, ESI Table S2.† The electronic energy ranges do overlap in 34 stoichiometries. However, the box-plot diagrams show the energy distributions are different and they should not be grouped, ESI, Fig. S5.†

The training and the test set must be representative of our dataset. For example, if none of the 4 heavy atom molecules have ended up in the test set, this is not a good training and test set split. Therefore, the molecules are assigned to the training and the test set by the following rules: (1) the algorithm loops over the unique stoichiometry and the number of molecules within each stoichiometry are extracted. (2) If the number of molecules is one, then the molecule is neither assigned to the training nor the test set. (3) If the number of molecules is two then one randomly selected molecule is assigned to the training set and the other molecule is assigned to the test set. (4) If the number of molecules is three or more, the molecules are assigned to training and the test set by setting test_size = 0.33 in train_test_split function in the scikit-learn module. Overall, 33.3% of the molecules (slightly more than 33.0% as expected) in the database are assigned to the test set.

We have tested a variety of different feature generation methods (please see ESI, Section 2.2† for more details). The performances of the different feature vectors are evaluated with neural network machine learning models. All neural network trainings are done with Keras^50^ and all the validations and the predictions are done with scikit-learn^51^ Python modules. Technical details about how we have set up the NN for training is described in detail in ESI, Section 2.1.† The full table of different representations and learning rates tested are shown in ESI, Table S3 and Fig. S8.†

The number of features is the sum of all KDE maxima for all bond, angle, dihedral and H-bonding KDE plots, which is determined by our choice of KDE widths. We have tested different number of features by varying the KDE widths (ESI, Table S8†). The best performing representation had 761 features. The representation which shows the best performance is when the angle connectivity information is included for the angle classifications, but the types of carbon information is not included for the dihedral classifications, Fig. S8.† Furthermore, the bond KDE bandwidths and the angle KDE bandwidths are set to 0.07. The best performing representation has the mean absolute error of 1.80 kcal mol^−1^.

### 3.2 ML models and hyperparameter search

The chosen representation has then been systematically tested with various other machine learning methods. We explored the multivariable linear regression (MLR), the kernel ridge regression (KRR)^52^ and the random forest regression (RFR).^53^ We also performed a further hyperparameter search for our present neural network model.

The KRR based machine learning models predicts the property of a molecule with representation ***M***, by the sum of the weighted kernel functions *K*(***M***, ***M***^train^_*i*_) between all the molecules in the training set (***M***^train^_*i*_) and ***M***. Specifically, the observable *O*(***M***) is predicted by 
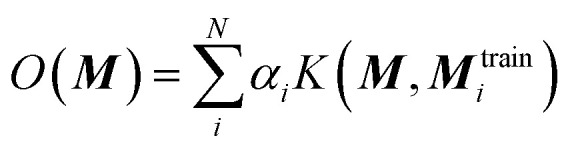
, where *α*_*i*_ are the weights and *N* is the number of molecules in the training set. The weights are found by minimising the Euclidean distance between the predicted and the reference molecules in the training set. We implemented the radial basis function (RBF) kernels from scikit-learn. The molecules in the database are randomly split into the training and the test set. 33% of the molecules are allocated to the test set. The RBF kernels have two hyperparameters that must be optimised: the variance (*σ*) and the regularisation (*α*). We have tested a wide range of *σ* and *α* for our hyperparameter search, ESI Fig. S9.†

The top performing KRR model has MAE of 1.23 kcal mol^−1^. Pople *et al.*^54^ have previously compared the accuracy of B3LYP energies of small organic molecules to experimental atomisation energies. The average absolute deviation from the experimental values is found be around 3.11 kcal mol^−1^. Lilienfeld *et al.* reports similar results with average absolute deviation of 2.5 kcal mol^−1^.^19^ Therefore, using our explainable feature set and database size around ∼57k molecules, KRR model achieve accurate results in the training set sized molecules. Strong regularisation is not necessary as the best performing model has *α* = 1 × 10^−11^. This is a consistent finding with Lilienfeld *et al.*^19^ Furthermore, very small kernel widths *σ* = 100 did not improve the model; the full hyperparameter search table is provided in ESI, Table S4.†

Further hyperparameter searching is necessary for the NN method to ensure the model from Section 2.1 is optimal. The choice of the NN architecture can potentially influence the performance of the model. We therefore varied the number of hidden layers and tested the model with 7 different learning rates, ESI, Table S5.† We have also tested different optimisers and regularisations, see ESI Section 2.3 (Table S6)† for full details. The best performing NN has MAE of 1.80 kcal mol^−1^. The predictive accuracy of the KRR method is superior to the NN method, when modelling molecules of same size as in the training set. This is a consistent with the results of Lilienfeld *et al.*^19^

The MLR is a simple but a useful approach to verify the robustness of our representation. We fitted the linear model by minimising the regularised ordinary least squares loss with stochastic gradient descent from scikit-learn. The regularisation factor is the hyperparameter that needs to be optimised. The training and the test sets are constructed by randomly allocating 33% of the molecules in the database to the test set and the remaining molecules to the training set. The hyperparameter search data is available in ESI, Table S7.† As expected, the MLR model does not perform well at predicting absolute electronic energies of these molecules with MAE of 25.73 kcal mol^−1^. Nevertheless, the results show our representation can predict the electronic energy to some degree even with a very simple machine learning approach. Importantly, this also allows evaluating each molecular feature's energetic importance. This in turn allows understanding molecular properties and reactivity of a very large variety of diverse molecules.

**Fig. 4:**
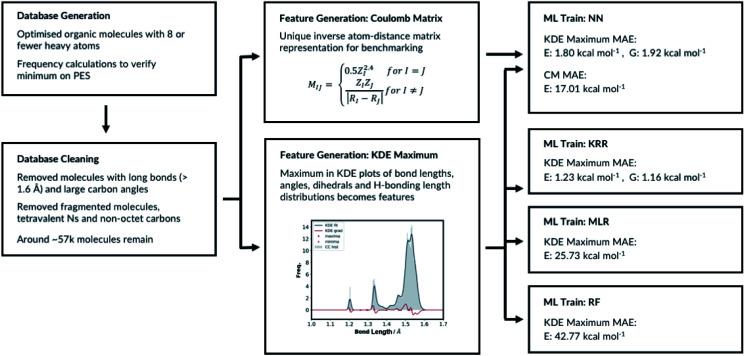
Summary of ML models training.

RFR is an ensemble learning method where the outputs of multiple decision trees are averaged to predict the observable. The decision trees are fitted on randomly sampled sets of training molecules. 33% of the molecules are randomly selected again and allocated to the test set and the remaining molecules are allocated to the training set. The RFR hyperparameters include the number of features to resample, the maximum number of samples to train the trees, the number of trees and the tree depth. We have implemented the RFR with the Python scikit-learn module and initially explored the maximum number of features and the maximum number of samples (ESI, Table S8†). The model performs best when maximum number of features is set to the number of features in the input node and the maximum number of samples is 100%. Altering the maximum tree depth and number of decision trees from the best performing model does not significantly affect the outcome, ESI Table S9.† The MAE of the best performing model is 42.77 kcal mol^−1^. Therefore, the RFR approach performs poorly compared to the MLR, the KRR and the NN models. All the different machine learning models we have tested, and their performances are summarised in Fig. 4.

### 3.3 Comparison with other features in the literature

**Fig. 5:**
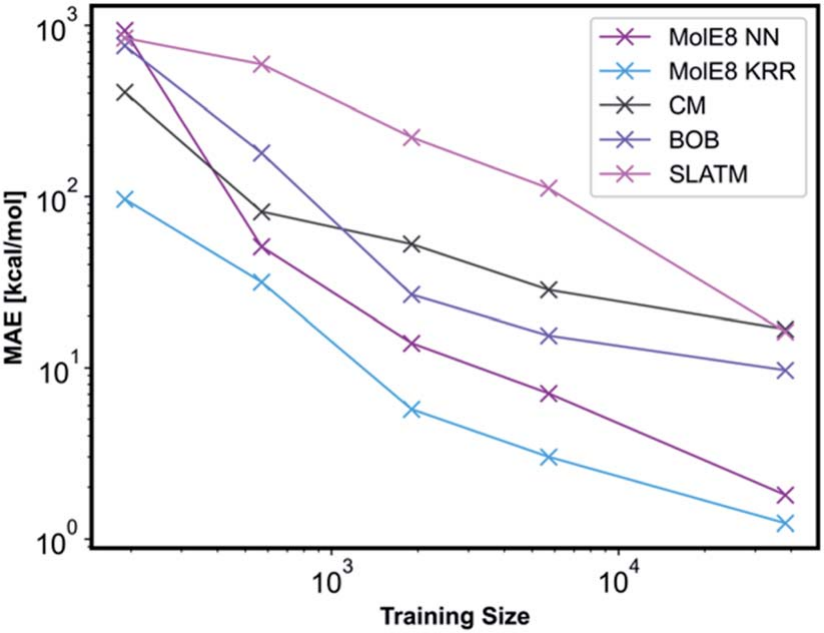
Performance of different representations as a function of training set sizes.

We have compared the accuracy of our representation with other representations in the literature, namely Coulomb matrix (CM), Bag of Bonds (BOB) and Spectrum of London and Axilrod-Teller-Muto (SLATM).^4^ These representation have been generated for all the molecules in our database using the QML Python library.^55^ All three representations were subsequently trained with NN and the technical details are outlined in ESI, Section 2.3, Tables S10–S13.† An informative method to compare the performance of representations is to plot learning curves.^4^ Therefore, we have measured the prediction performance of CM, BOB, SLATM, MolE8 representations as a function of number of training points. We have generated four new training databases containing 190, 570, 1900 and 5700 molecules randomly selected from the ∼38 000 molecule training set. The ML models trained from these datasets using the mentioned representations have subsequently been used to predict the electronic energy of the molecules in the original ∼ 19 000 test set, Fig. 5.

For the CM representation, the best performing model has MAE of 17.01 kcal mol^−1^, which is almost order of magnitude worse than the NN approach with our new representation. This MAE is higher than the value reported in the literature by Lilienfeld *et al.*^19^ However, the number of training molecules in our database is significantly smaller than the Lilienfeld's dataset and therefore the algorithm has to make predictions by exploring a smaller chemical space.

Across all training set sizes, the MolE8 KRR model has the best performance. The MolE8 NN model has superior performance to other representations when there are more than 570 molecules in the database. Furthermore, the MAE do not appear to have converged for both MolE8 methods, meaning the MAE can be lowered even further if they are trained on larger databases. In contrast, BOB and CM representation error plots might be showing signs of flattening out. This emphasises that our developed features are much more data-efficient than the previous approaches, as they are able to achieve superior performance even at significantly smaller dataset size.

### 3.4 Free energy predictions

All the results so far focused on the prediction of electronic energy. We also explored the performance of the KRR and the NN approaches on the free energy predictions. For the NN model training, if the two molecules have the same free energy values, only one of the two molecules was included for training. This resulted in removal of further 286 molecules in the database. The remaining database cleaning steps are the same as the electronic energy prediction section. The atomisation free energies at 298.15 K are extracted from the Gaussian 16 frequency calculations as the target output. The molecules are allocated to the training and the test set using the stoichiometry grouping described earlier. The distributions of bond lengths, angles, dihedrals and H-bond lengths are plotted as before. The bandwidths for the bond and angle plots are kept the same. The KDE maxima are then used to generate the features. Since few more molecules have been removed in the new database, the number of automatically generated features from KDE is different from the electronic energy prediction section. 781 features have been created from the KDE plots.

The NN architecture is identical to the electronic energy prediction model with two hidden layers with weights and bias initialised from the random uniform distributions, the mean squared error loss function and the training batch size of 64. The ReLU activation is used for the hidden layers and the linear activation is used for the output layer. We tested the Adam, SGD and RMSprop optimisers and four *L*_2_ regularisation factors ranging from 0.05 to 0.20 as the hyperparameter search, ESI Table S14.† The best performing model used the Adam optimiser and has *L*_2_ the regularisation factor of 0.15. The MAE of the best performing model is 1.92 kcal mol^−1^, which is a similar value to the electronic energy prediction NN. Importantly, the MAE is still below the expected average deviation error of B3LYP method from the experimental values.

For the KRR training, the method is identical to the electronic energy prediction model. 33% of the molecules in the database are randomly allocated to the test set and the remaining molecules are allocated to the training set. Many different variances and the regularisation values have been tested as the hyperparameter search, ESI Table S15.† The MAE of the best performing model is 1.18 kcal mol^−1^, a similar value to the electronic energy prediction KRR model and superior accuracy compared to the NN approach. Therefore, our representation is able to predict the free energy of the organic molecules in the database.

### 3.5 Comparison with semi-empirical methods

Semi-empirical quantum chemistry methods are classical baselines that are still used today for quick and efficient calculations. Therefore, we have compared the performance of MolE8 to PM7 semi-empirical approach.^56^ Our algorithm loops over all the structures in the database and if the molecular formula is C_6_H_9_NO and is present in the test set, we generate the input file for Gaussian 16 PM7 calculation. C_6_H_9_NO is a very frequently occurring molecular formula in our database with over 2300 molecules in total and around 780 molecules in the test set. We have optimised the PM7 structures with Intel Xeon Skylake 6142 processors, 2.6 GHz 32-cores in total. The total calculation time was 5 hours.

We then compared the relative PM7 and MolE8 energies of all the isomers of C_6_H_9_NO in the test set to the relative DFT electronic energy. The PM7 method predicts the relative energies of the isomers with the MAE of 5.99 kcal mol^−1^. Using our KRR and NN models, the relative energies of the isomers have been predicted with the MAE of 1.69 kcal mol^−1^ and 3.36 kcal mol^−1^, respectively. The MolE8 calculations were performed using 3.1 GHz Intel Core i7 4-cores in total. The total calculation time was around 4 seconds. Therefore, MolE8 predicts the relative electronic energy of C_6_H_9_NO isomers with 3.5 times lower MAE with an efficient computation time.

### 3.6 Training with distorted molecules

**Fig. 6:**
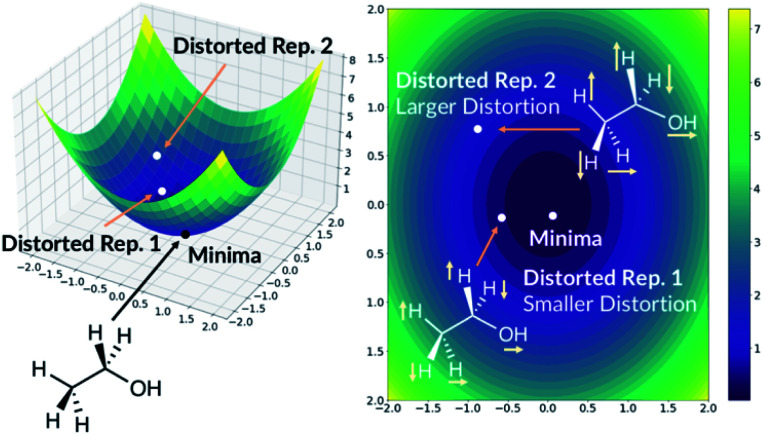
Adding distorted molecule replicates to the database. The axis are arbitrary.

The quick molecular energy prediction would have a wider application if the molecule did not have to be optimised with DFT methods in advance. We therefore explored the possibility of our ML methods to make energy predictions on molecules that are not precisely on the DFT minima, but close to it on the potential energy surface. For every molecule in the database, we replicate the structure and translate every atom by a constant amount in randomly selected *x*-axis, *y*-axis or *z*-axis direction. These structures are then added back to the original database. The database will now contain the minima on the potential energy surface but also the points nearby the minimum, Fig. 6. The target outputs are the energies of the true DFT minimum for both minimum and off-minimum structures. The identical feature generation method is then used for ML training.

For NN models, we first explored the effect of including multiple distorted molecule replicates. Three new databases have been created with one, two and three distorted molecule replicates respectively. The degree of distortion is set to 0.01 Å. The molecules in the database are then split into the training and the test set. When the molecules in the new database are allocated, we ensured that the molecules in the test set in Section 2.2 are also assigned to the present test set. Therefore, the new test set contains the DFT optimised geometries and the distorted geometries. After training the NN models, we concluded that including the distorted molecule replicates improve the model since the MAE decreased to 1.67 kcal mol^−1^ (ESI Section 2.6†). However, including more distorted molecule replicates with the same degree of distortion does not improve the model. We also explored the databases with different combination of distorted molecule replicates. Three new databases are created with two distorted molecule replicates where the degree of distortion is set to [0.01 Å, 0.05 Å], [0.01 Å, 0.10 Å] and [0.05 Å, 0.10 Å]. After training the NN models, we found that MAE increases as the degree of distortion increases, ESI Section 2.6.† Therefore, [0.01 Å, 0.05 Å] is a compromise between low test set MAE of 1.70 kcal mol^−1^ (similar to the MAE when the database only contained 0.01 Å distorted molecule replicates) and the size of the basin from the potential energy surface minima. When the best performing NN model trained without the distorted molecule replicates is applied to the present test set with the distorted molecule replicates, the MAE radically increases to 72.54 kcal mol^−1^, indicating that the original model is very sensitive to moving away from the minima geometry. Therefore, the NN trained with distorted molecule replicates is much more robust and can now predict the electronic energy of the DFT PES minima when the molecular structure is near the minima.

**Fig. 7:**
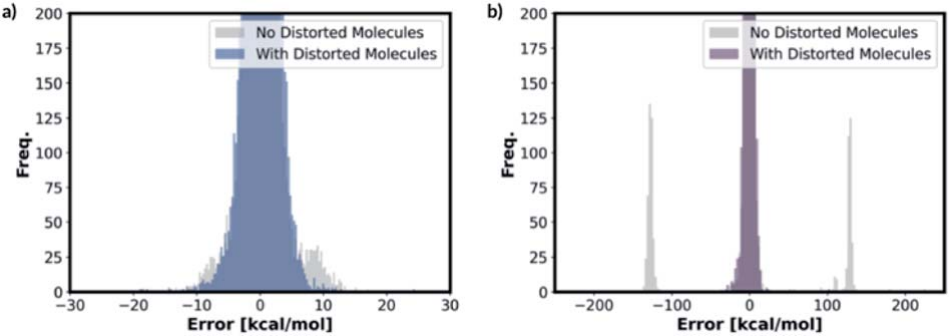
Distribution of errors in (a) KRR and (b) NN models when the model trained with and without the distorted molecule replicates is used to predict the test set energies containing distorted molecule replicates (for both KRR and NN models, we have predicted the energy of the one 0.01 Å distorted molecule replicate database for this figure).

We also trained the databases with different combinations of distorted molecule replicates with KRR methods. The MAE of 1.23 kcal mol^−1^, for one distorted molecule replicate with 0.01 Å distortion, is almost identical performance to the best performing KRR model from Section 2.2, Table S16.† When the best performing KRR model trained without the distorted molecule replicates is applied to the test set with the distorted molecule replicates, the MAE increases slightly to 1.44 kcal mol^−1^. Therefore, training with the distorted molecule replicates has improved the ability of the KRR model to predict PES minima energy when the structure is close to the minima, but not as significantly as the NN model. We have plotted the distribution of the errors when the test set containing the distorted molecule replicates are modelled with the NN and KRR methods trained with and without the distorted molecule replicates. The distribution of the errors for the NN model is very spread out when NN is trained without the distorted molecule replicates, Fig. 7. However, the inclusion of the distorted molecule replicates in NN training reduces the spread and hence the significantly reduced test MAE. The distribution of the errors for the KRR model trained without the distorted molecule replicates is already very centred around 0.0. Therefore, the reduction in MAE is significantly less than the NN model when the distorted molecule replicates are included in the training.

To further test the applicability of the ML models trained on distorted molecule replicates, we take the DFT optimised molecules in the test set and re-optimise them using the MMFF method from RDKit. Around 2000 molecules have been removed because RDKit failed to generate the molecule object from the DFT optimised structure. Further 646 molecules have been removed because the InChI before the MMFF optimisation does not match the InChI after. The DFT energies of MMFF optimised molecules are predicted well with one 0.01 Å distorted molecule replicate trained KRR model with MAE of 2.50 kcal mol^−1^. The KRR models trained with two distorted molecule replicates have superior performance when modelling the MMFF optimised test set molecules but they require a large amount of memory. We therefore recommend the one 0.01 Å distorted molecule replicate trained KRR model for the routine usage. This model does not use as much memory as two distorted molecule replicates trained models but still has lower MMFF MAE than the KRR model trained without it.

Moreover, the distortion needs to be sufficiently large such that adequate region around the PES minima has been sampled. In order to quantify how much the representations change due to the distortions, we define the distortion constant as below:1
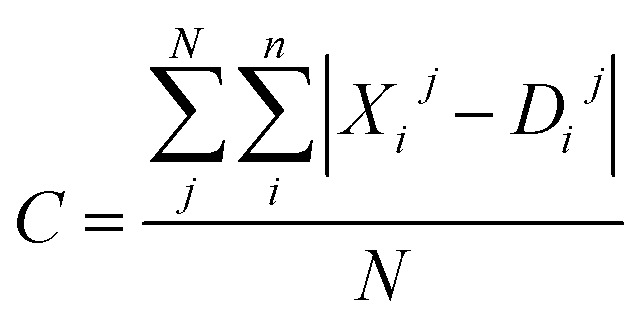
where *X*^*j*^ is the features vector, *D*^*j*^ is the distorted features vector, *n* is the number of features and *N* is the number of samples. For the datasets with one distorted molecule replicates of 0.0005 Å, 0.01 Å, 0.05 Å and 0.1 Å, we have found the distortion constant to be 3.49, 6.89, 23.72 and 36.23, respectively. We have then applied the KRR model trained with one distorted molecule replicate to predict DFT energy from the MMFF optimised test set, ESI Table S18.† From the result, we conclude that *C* = 6.89 is the best balance between the test set MAE and the MMFF test set MAE.

When the NNs trained with distorted molecule replicates are used to predict the MMFF optimised test set molecules, the best performing models have similar MAEs to the KRR models with 2.46 kcal mol^−1^. Adding additional distorted molecule replicates of 0.01 Å do not decrease the MAE significantly. [0.01 Å, 0.05 Å] distorted molecule replicate trained NN is again the best compromise between the DFT test set MAE and the MMFF test set MAE.

**Fig. 8:**
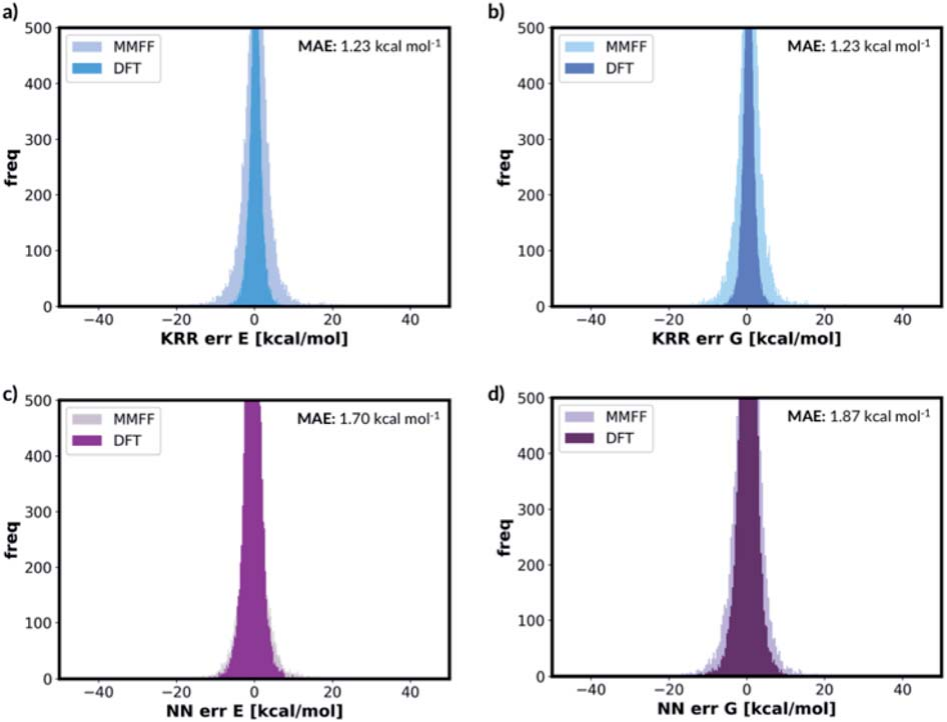
Error distribution of different ML models trained with distorted molecule replicates. ‘MMFF’ and ‘DFT’ means MMFF and DFT optimised test set error distribution, respectively. (a) KRR trained model for electronic energy, (b) KRR trained model for free energy, (c) NN trained model for electronic energy, (d) NN trained model for free energy.

For free energy predictions, we have trained two KRR models with one 0.01 Å and one 0.10 Å distorted molecule replicates, respectively. The 0.01 Å distorted molecule replicate trained model again has better compromise the test set (1.23 kcal mol^−1^) and MMFF test set (2.38 kcal mol^−1^) MAEs. We have also trained the NN model with two distorted molecule replicates (0.01 Å and 0.05 Å) for the free energy prediction with the test set MAE of 1.86 kcal mol^−1^ and MMFF test set MAE of 2.57 kcal mol^−1^. When the best performing NN trained without the distorted molecule replicates is used to predict the free energy for the test set containing the distorted molecule replicates, the MAE increases to 73.78 kcal mol^−1^. When the KRR trained without the distorted molecule replicates is used to predict the free energy of the test set containing 0.01 Å distorted molecule replicates, the MAE increases to 1.34 kcal mol^−1^ from 1.23 kcal mol^−1^. The errors in predicted energies and free energies of DFT and MMFF optimised structures using the distorted molecule replicates trained models is summarised in Fig. 8. KRR method shows narrower DFT errors but NN has superior transferability when predicting MMFF structure energies. Overall, this 3D geometry data augmentation technique has improved the performance and robustness of many ML approaches we have investigated and could potentially be useful in other contexts.

### 3.7 Extrapolation to molecules with 9 or more heavy atoms

To assess the transferability of MolE8.py, we constructed four new test sets containing the molecules with number of heavy atom counts between 9 and 12. For the 9-atom test set, around ∼1k molecules are randomly selected from the list of SMILES strings of GDB13 (ref. 23) database. The database clean-up procedure described for 8 or fewer heavy atom molecules is then applied to the 9-atom test database. 1115 test molecules remained in the database after the cleaning step. The random selection and the clean-up steps are then repeated for 10, 11 and 12-atom test databases from 10, 11 and 12 heavy atom list of strings in the GDB13, respectively. The final 10-atom test set contains 1107 molecules, the 11-atom test set has 1095 molecules and the 12-atom test set has 1060 molecules.

**Fig. 9:**
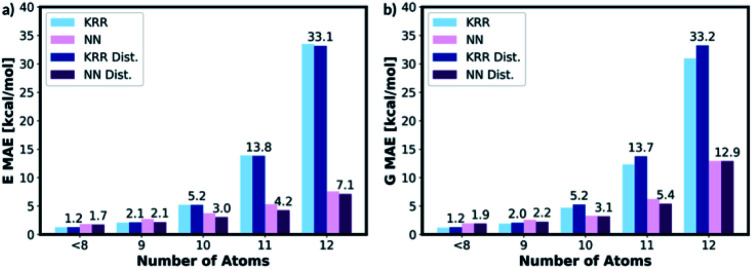
KRR and NN models trained with and without the distorted molecule replicates have been used to predict energies of the molecules in the 9–12 heavy atoms databases. The KRR model has superior performance for 9 or fewer heavy atom molecules and the NN model has superior performance for 10 or more heavy atom molecules.

The representation is then generated for all the molecules in the 9, 10, 11 and 12 atom test databases. The trained KRR and NN models (with and without the distorted molecule replicates) for the 8 or fewer heavy atom database are then imported and the energy and the free energy predictions are made on the new test databases, Fig. 9. For 9 or fewer atom molecules the KRR models have the lowest MAEs. The energies and the free energies are predicted with 2.06 kcal mol^−1^ and 1.89 kcal mol^−1^ MAEs, respectively for 9 atom molecules. The difference in MAE between KRR trained with and without the distorted molecule replicates is less than 0.15 kcal mol^−1^. For 10 or more heavy atom molecules, the NN models have superior performance to KRR. Furthermore, the MAE of the NN trained with the distorted molecule replicates is always lower than the NN trained without. Therefore, for 10 or more heavy atom molecules, we recommend NN trained with the distorted molecule replicates which has superior extrapolatability.

The poor performances of the KRR approaches for out-of-sample molecules have been mentioned in the literature.^39^ The observable is predicted from the weighted sum of the kernel functions constructed from the molecules in the training set. Therefore, the 10 or more heavy atom test set data points are very far away from the training set data points and the weighted sum becomes increasingly inaccurate. The NN approaches seem more compatible with ‘higher number of atoms’ extrapolation.^27^ Therefore, we recommend that the users of MolE8.py to select the KRR model for 9 or fewer heavy atom molecule and NN model for 10 or more heavy atom molecule energy predictions.

Furthermore, we have listed the 9 and 10 heavy atom molecules that have largest MAEs for KRR and NN predictions, ESI Fig. S11 and S12.† Many molecules have ring systems that are impossible for an 8 heavy atom molecule. For example, the 6–5 fused ring or 6-membered ring with 3 substituents are not possible within 8 heavy atoms. Furthermore, molecules with long conjugated systems also often have large errors. Long conjugated systems are expected to be a problem since accounting for it requires global understanding of the molecule whereas our present representation only considers the local bonds, angles and dihedrals.

### 3.8 Comparison to other ML approaches in the literature

We have compared the performance of MolE8 to other open-source ML approaches in the literature, namely PhysNet^57^ and SchNet.^58^ SchNet is neural network potential constructed from symmetrised gradient-domain machine learning to achieve *ab initio* level molecular dynamics simulations. PhysNet is more recent neural network potential designed on physical principles inspired from SchNet. Both approaches have state-of-the art accuracy for electronic energy prediction on the QM9 dataset with the combined training and test sample size around 50 000. PhysNet and SchNet achieves MAE of 0.30 and 0.49 kcal mol^−1^.^57^ However, the ML models have been trained on NVIDIA GeForce GTX TITAN GPUs for 1–2 days to achieve these results and therefore are not computationally cheap.

To compare the computational efficiency, we have trained PhysNet and SchNet to the same training set we have used to train MolE8. All three models have been trained at the same computational level using Intel Xeon Skylake 6142 processors, 2.6 GHz 32-cores in total. The MolE8 KRR model only took 5 minutes train. However, the PhysNet and SchNet errors have not converged after 20 hours when trained without the GPUs. Our NN method approach also has faster convergence to PhysNet and SchNet. We have plotted the convergence of MAE against the training time in ESI, Section 2.9.† Therefore, MolE8 approaches have state-of-the-art data efficiency for its accuracy.

Finally, we have compared the speed of our algorithm to TorchANI program by Roitberg *et al.*^28^ The time taken by our algorithm to calculate energies of the molecules in the 9–12 atoms database is up to 7 times faster than TorchANI program and recorded 1000 energy calculations per second on a standard 8-core Intel i7 CPU, ESI Section 2.7.† This is expected since ANI-1 is a more complete model of the potential energy surface, while our model is designed for rapid estimation of energies at optimum. We believe our algorithm will have potential applications where many DFT energy calculations are required, for example, when rapid and accurate energy calculations are needed from an ensemble of force-field optimised molecules.

## 4 Conclusions

We present a new representation for organic molecules which can be used to build machine learning models to predict the ground state electronic energies and the free energies. All the bond lengths, angles, dihedrals and H-bonding lengths of the molecules in the database have been collected and the distributions these molecular properties are plotted. The maxima in the distributions become the features. Our representation counts the number of occurrences of these maxima along with the atom types of the molecule in question. This representation has been coupled with the kernel ridge regression, the neural network, the multivariable linear regression and the random forest regression machine learning models. The optimised kernel ridge regression electronic energy prediction model has the best performance for 8 or fewer heavy atom organic molecules with the mean absolute error of 1.23 kcal mol^−1^, followed by the neural network model with the mean absolute error of 1.80 kcal mol^−1^. For the free energy prediction machine learning methods, the kernel ridge regression model and the neural network model have mean absolute error of 1.18 and 1.92 kcal mol^−1^, respectively. We have also trained neural network and kernel ridge regression models with distorted molecule replicates to sample geometries near the DFT potential energy surface minima. The distorted molecule replicate trained kernel ridge regression and neural network models can predict the DFT energies using the MMFF optimised geometries for 8 or fewer heavy atom molecules with mean absolute error of 2.50 and 2.46 kcal mol^−1^, respectively. We have also shown that our methods are extrapolatable to molecules with number of heavy atoms greater than 8. The performance of the kernel ridge regression model is the best up to 9 heavy atom molecules, but the neural network performs better for 10 or more heavy atom molecules. Furthermore, the neural networks trained with the distorted molecule replicates always have superior performance, thus demonstrating the benefits of this data augmentation. The algorithm is computationally very cheap and can predict the energies for multiple molecules instantaneously. Overall, our model combines state-of-the-art data efficiency and extrapolatability with data-efficiency, explainability and ground-breaking computational efficiency. For these reasons, we expect this method to find diverse applications in many areas.

## Data availability

The latest version of MolE8.py script is available for download from University of Cambridge Repository (https://doi.org/10.17863/CAM.78009) and the full set of Gaussian output files used in the training is available from University of Nottingham repository (https://doi.org/10.17639/nott.7159). The program is also available on GitHub: https://github.com/sanha0213/MolE8.

## Author contributions

All authors have contributed equally. The manuscript was written through contributions of all authors. Everyone involved in the manuscript have given approval to the final version of the manuscript.

## Conflicts of interest

There are no conflicts of interest.

## Supplementary Material

SC-013-D1SC06324C-s001
