# Supplementary material for: MolE8: finding DFT potential energy surface minima values from force-field optimised organic molecules with new machine learning representations
Source: Chem Sci. 2022 May 28;13(24):7204–14. doi: 10.1039/d1sc06324c (PMC9214916; doi:10.1039/d1sc06324c)
Supplement: SC-013-D1SC06324C-s001 [file SC-013-D1SC06324C-s001.pdf]

Electronic Supplementary Material (ESI) for Chemical Science.
This journal is © The Royal Society of Chemistry 2022

Supporting Information

MolE8.py: Instantaneous DFT Energy Calculations from 8-heavy atoms Database of Small Organic Molecules with Machine Learning

Sanha Lee, Kristaps Ermanis, Jonathan M. Goodman

Department of Chemistry, University of Cambridge
Lensfield Road, Cambridge, CB1 1EW

Table of Contents

1. DATABASE CLEANING 3

1.1 Removed Non-Octet Carbon Molecule Examples 3
1.2 Removed Example Molecules with Large Angles 3
1.3 Molecules with Long Bonds 4

2. SELECTING THE OPTIMAL REPRESENTATION 5

2.1. Molecular formulas present in the database 5
2.2. Testing different feature generation for NN models 14
2.3. ML Models and Hyperparameter Search 16
2.4 Free energy predictions 20
2.5. Extrapolation to molecules with 9 or more heavy atoms 21
2.6. Training with distorted molecules 23
2.7. Extrapolation to 9 or more heavy atoms 24

1. Database Cleaning

1.1 Removed Non-Octet Carbon Molecule Examples

The structures below are 10 examples out of 75 molecules. All the hydrogens in the molecules are drawn explicitly.

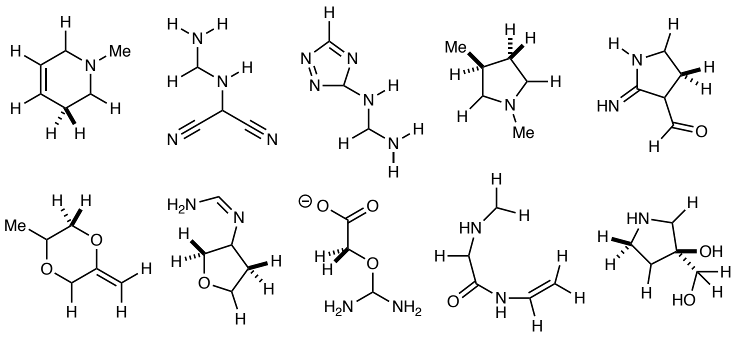

Figure S1. Removed example non-octet carbon molecules

1.2 Removed Example Molecules with Large Angles

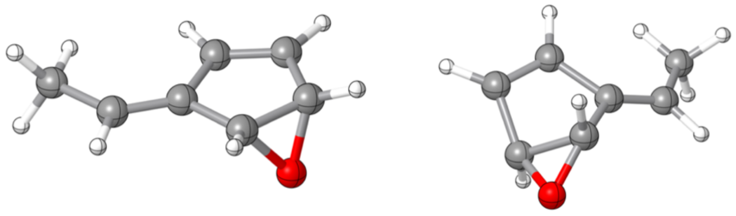

cis-expoxide
E = 0.0 kcal mol^{-1}

trans-expoxide
E = 82.9 kcal mol^{-1}

Figure S2. cis-epoxide and trans-epoxide comparison.

During our initial investigation, we found the neural network models performed poorly on molecules containing trans-epoxide motifs. We optimised two structures shown Figure S2 to compare the cis and trans epoxide structures. The trans-epoxide is substantially less stable than the cis-epoxide by 82.9 kcal mol^{-1}. Therefore, we felt it is reasonable to remove the trans-epoxide structures from the database.

The trans-epoxide motif is characterised by large angle around the carbon atom. All the angles around the two carbon atoms forming the epoxide motif in the two structures above are shown in Table S1. The angle in red is significantly larger in the trans motif compared to the cis motif.

Table S1. cis and trans epoxide carbon angle comparison. C1 and C2 are two carbons forming the epoxide motif. All angles are in degrees and Diff. is 109.5°-Angle.

cis						trans					
**C1**	**Angle**	**Diff.**	**C2**	**Angle**	**Diff.**	**C1**	**Angle**	**Diff.**	**C2**	**Angle**	**Diff.**
CCC1	106.9	2.6	CCC2	106.1	3.4	CCC1	96.3	13.2	CCC2	95.7	13.8
CCH1	121.2	11.7	CCH2	124.2	14.7	CCH1	109.0	0.5	CCH2	109.7	0.2
HCO1	115.3	5.8	HCO2	115.3	5.8	HCO1	107.0	2.5	HCO2	106.5	3.0
OCC1	50.4	59.1	OCC2	59.1	50.4	OCC1	58.6	50.9	OCC2	57.6	51.9
CCO1	114.4	4.9	CCO2	113.1	3.6	CCO1	142.2	32.7	CCO2	142.0	32.5
HCC1	124.4	14.9	HCC2	124.2	14.7	HCC1	113.2	3.7	HCC2	115.1	5.6

We reasoned more highly strained molecules could be identified by looking at the angle around the carbon atom. The critical large angle was chosen by investigating different connectivity carbon motifs, Figure S3. For 3 connectivity carbon atoms, we remove the molecule if [carbon angle – 120°] is larger than 35° or [360° - sum of three carbon angles] is larger than 20°. For four connectivity carbon atoms, we found carbon angle larger than 29° is a reasonable cutoff point.

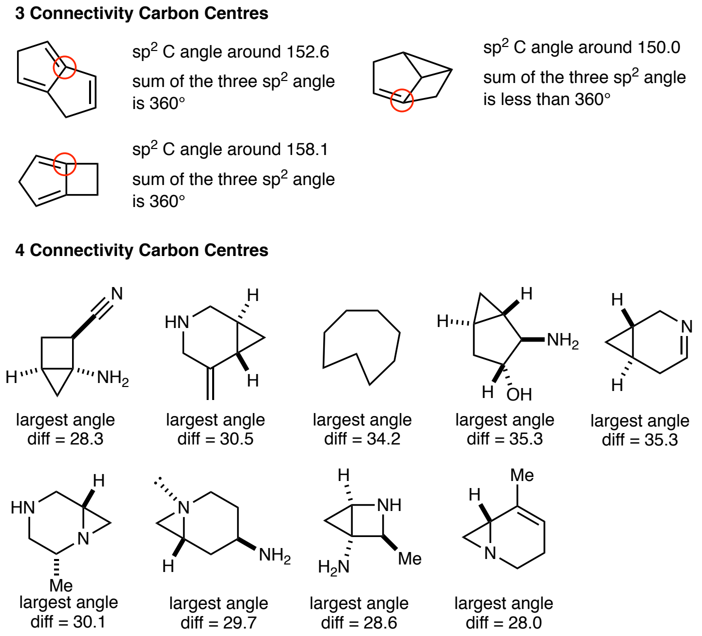

Figure S3. Different structural motifs for 3 connectivity and 4 connectivity molecules with large carbon angles

Overall, the molecules satisfying the following conditions were removed from the database:

- The molecule has 4 connectivity carbon with its largest angle greater than 29°
- The molecule has 3 connectivity carbon with 360 – sum(angles) is larger than 20°
- The molecule has 3 connectivity carbon with its largest angle greater than 35°
- The molecule has 2 connectivity carbon with 180 – angle greater than 20°

1.3 Molecules with Long Bonds

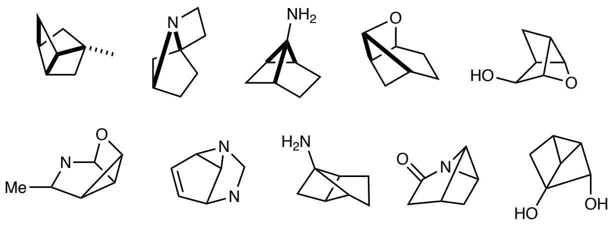

Figure S4. Removed Long Bond Molecules

2. Selecting the Optimal Representation

2.1. Molecular formulas present in the database

Table S2. Every unique molecular formula present in the database. Min_E and Max_E are the minimum and the maximum energy molecule in the database, Mean_E is the mean energy of the molecules of a particular molecular formula and N_Samples is the number of molecules a particular molecular formula contains.

Idx	Mol_Forms	Min_E	Max_E	Mean_E	N_Samples
0	C4N2O2H12	-263313.32	-263304.57	-263309.61	5
1	C1N0O2H2	-119085.82	-119085.82	-119085.82	1
2	C5N2O0H14	-193579.87	-193563.76	-193573.84	35
3	C4N1O1H3	-178319.49	-178280.76	-178307.40	7
4	C5N2O1H6	-237772.07	-237641.19	-237717.33	1021
5	C3N5O0H9	-246944.02	-246927.76	-246936.72	6
6	C4N0O1H10	-146646.24	-146635.67	-146640.54	7
7	C4N0O2H6	-192342.24	-192258.18	-192306.82	54
8	C0N5O0H1	-172112.16	-172112.16	-172112.16	1
9	C6N0O2H6	-240160.71	-240031.36	-240105.60	479
10	C5N2O0H4	-189781.88	-189681.72	-189737.21	25
11	C2N2O3H2	-258913.04	-258858.59	-258887.20	7
12	C6N1O0H13	-182762.68	-182727.48	-182739.32	193
13	C2N2O1H4	-165253.98	-165249.37	-165251.94	3
14	C5N1O0H7	-156568.22	-156474.91	-156523.04	38
15	C1N4O0H2	-162067.77	-162065.44	-162066.60	2
16	C4N0O2H4	-191571.23	-191458.95	-191531.60	19
17	C2N3O2H5	-247236.40	-247236.40	-247236.40	1
18	C2N5O0H1	-219946.79	-219946.79	-219946.79	1
19	C6N0O0H10	-147255.60	-147209.03	-147234.31	35
20	C7N1O0H17	-208190.11	-208171.52	-208184.28	88
21	C4N0O3H4	-238784.21	-238700.97	-238749.65	26
22	C6N0O1H14	-195988.85	-195978.57	-195983.54	32
23	C3N2O2H4	-236409.18	-236319.22	-236359.54	56
24	C3N1O1H3	-154422.42	-154351.95	-154400.28	7
25	C5N1O2H13	-253253.29	-253231.58	-253240.87	96
26	C5N2O1H10	-239295.06	-239189.69	-239239.67	1853
27	C5N1O0H11	-158085.77	-158057.29	-158066.73	62
28	C5N3O0H3	-223758.05	-223674.63	-223713.83	35
29	C4N1O2H9	-227853.90	-227767.78	-227807.90	128
30	C3N2O3H2	-282848.25	-282726.55	-282789.68	54
31	C4N2O1H10	-215383.84	-215302.97	-215336.01	183
32	C2N2O0H8	-119563.07	-119563.07	-119563.07	1
33	C3N1O4H3	-296080.39	-296010.54	-296043.59	12
34	C4N0O2H8	-193109.47	-193050.35	-193072.23	48
35	C3N5O0H5	-245449.64	-245349.28	-245394.57	52
36	C4N0O4H8	-287513.10	-287460.54	-287494.91	17
37	C3N0O1H8	-121972.12	-121964.04	-121968.26	3
38	C7N0O0H6	-169598.71	-169550.61	-169569.31	17
39	C6N2O0H2	-212879.37	-212870.47	-212873.82	4
40	C5N0O0H6	-121809.61	-121770.64	-121783.63	5
41	C6N0O0H8	-146485.52	-146422.60	-146457.51	30
42	C2N3O0H3	-152023.95	-152007.44	-152015.33	6
43	C2N0O4H2	-237424.62	-237424.62	-237424.62	1
44	C4N1O0H3	-131098.58	-131085.11	-131091.84	2
45	C6N2O0H6	-214458.80	-214314.66	-214401.46	290
46	C4N4O0H14	-238374.04	-238371.54	-238372.79	2
47	C4N1O1H11	-181380.30	-181359.14	-181370.98	18
48	C5N0O3H4	-262695.61	-262570.20	-262648.35	77
49	C3N2O3H4	-283611.84	-283522.96	-283568.13	49
50	C8N0O0H8	-194321.09	-194202.56	-194249.82	134
51	C4N0O3H8	-240312.95	-240253.21	-240283.22	43
52	C1N7O0H3	-265500.97	-265500.97	-265500.97	2

53	C4N1O3H5	-273549.13	-273397.20	-273500.91	171
54	C1N2O1H4	-141368.68	-141368.68	-141368.68	1
55	C6N1O1H9	-228459.40	-228336.30	-228403.22	2374
56	C4N3O0H1	-199049.14	-199049.14	-199049.14	1
57	C5N3O0H15	-228314.67	-228296.22	-228306.53	32
58	C4N0O4H6	-286772.78	-286704.01	-286737.25	50
59	C4N0O3H2	-237981.13	-237977.02	-237979.07	2
60	C4N4O0H12	-237629.52	-237592.94	-237618.78	30
61	C4N2O0H4	-165875.11	-165795.11	-165843.48	10
62	C6N0O0H14	-148786.56	-148784.88	-148785.71	5
63	C6N1O0H11	-181998.69	-181929.67	-181963.29	307
64	C4N2O1H6	-213859.09	-213745.44	-213810.47	295
65	C2N1O0H3	-83310.33	-83310.33	-83310.33	1
66	C4N0O0H2	-96320.97	-96320.97	-96320.97	1
67	C4N3O1H11	-250117.58	-250059.47	-250089.91	68
68	C3N4O0H8	-212200.82	-212187.81	-212193.93	11
69	C4N4O0H2	-233819.10	-233725.11	-233776.70	9
70	C7N1O0H15	-207435.10	-207395.45	-207411.27	588
71	C3N4O1H4	-257915.70	-257791.05	-257862.97	125
72	C3N1O1H5	-155192.20	-155136.43	-155171.65	14
73	C4N2O1H12	-216109.22	-216096.96	-216103.92	16
74	C3N1O0H7	-108725.67	-108713.19	-108719.50	5
75	C2N2O2H4	-212495.91	-212477.01	-212482.48	5
76	C4N1O3H3	-272778.64	-272682.98	-272736.64	77
77	C1N4O3H0	-302832.28	-302824.56	-302828.42	2
78	C2N4O1H2	-233184.67	-233122.01	-233166.24	8
79	C2N5O1H5	-268656.98	-268656.98	-268656.98	1
80	C3N0O0H6	-73995.07	-73987.17	-73991.12	2
81	C3N0O5H2	-308530.05	-308509.59	-308519.82	2
82	C4N4O0H10	-236875.95	-236835.77	-236858.29	77
83	C2N1O2H1	-176959.01	-176959.01	-176959.01	1
84	C6N1O1H13	-229983.52	-229920.27	-229940.69	1159
85	C4N0O0H4	-97106.05	-97106.05	-97106.05	1
86	C2N4O0H2	-185961.20	-185941.15	-185952.21	3
87	C4N3O0H7	-201380.29	-201314.01	-201356.58	107
88	C2N2O1H6	-166036.66	-166011.23	-166022.42	4
89	C4N2O1H4	-213093.57	-213012.01	-213055.88	96
90	C5N0O2H12	-218518.71	-218499.20	-218509.84	37
91	C4N1O1H5	-179105.87	-178994.87	-179068.05	53
92	C4N1O0H5	-131892.62	-131827.82	-131872.09	6
93	C5N0O2H8	-217017.45	-216924.02	-216973.49	314
94	C5N3O0H9	-226055.71	-225941.60	-226009.98	1097
95	C3N1O0H5	-107982.06	-107936.76	-107959.41	2
96	C6N0O0H2	-144115.21	-144115.21	-144115.21	1
97	C8N0O0H18	-198129.99	-198123.37	-198126.85	18
98	C6N1O1H7	-227702.93	-227555.50	-227637.86	1094
99	C7N1O0H1	-202004.34	-202004.34	-202004.34	1
100	C4N2O2H6	-261083.98	-260919.31	-261016.38	916
101	C3N0O2H6	-168437.75	-168385.54	-168407.48	11
102	C4N2O2H10	-262585.09	-262475.68	-262532.98	325
103	C6N0O2H14	-243191.01	-243171.54	-243181.64	107
104	C7N1O0H3	-202789.84	-202774.37	-202780.85	7
105	C3N3O0H9	-178222.30	-178184.38	-178212.43	13
106	C4N2O0H8	-167394.85	-167324.26	-167365.99	63
107	C3N2O1H10	-191434.75	-191434.65	-191434.70	2
108	C1N3O1H1	-174516.18	-174505.90	-174511.04	2
109	C5N0O3H2	-261889.75	-261860.83	-261872.39	3
110	C4N0O2H10	-193845.89	-193827.00	-193837.81	13
111	C3N1O2H7	-203179.05	-203118.99	-203156.87	15
112	C1N6O1H2	-277982.16	-277982.16	-277982.16	1
113	C4N4O0H4	-234592.69	-234515.21	-234564.09	65
114	C7N1O0H7	-204385.70	-204257.29	-204324.31	225
115	C2N4O2H0	-279559.24	-279559.24	-279559.24	1

116	C3N1O0H3	-107205.61	-107205.61	-107205.61	1
117	C6N0O0H12	-148029.03	-148004.14	-148011.00	25
118	C5N1O1H11	-205311.74	-205247.06	-205269.61	294
119	C7N1O0H5	-203634.53	-203473.94	-203554.13	35
120	C3N0O5H4	-309301.25	-309301.25	-309301.25	1
121	C1N0O1H4	-72621.90	-72621.90	-72621.90	1
122	C5N2O1H12	-240055.43	-239969.19	-240005.33	737
123	C4N3O1H1	-246266.25	-246239.54	-246255.07	5
124	C3N1O2H9	-203903.99	-203901.66	-203902.82	2
125	C3N1O2H1	-200844.23	-200844.23	-200844.23	1
126	C3N1O4H1	-295261.73	-295256.95	-295259.34	2
127	C8N0O0H10	-195097.15	-194976.23	-195032.08	354
128	C4N3O1H13	-250843.15	-250836.13	-250840.06	4
129	C5N0O3H8	-264227.76	-264123.34	-264183.74	408
130	C4N3O0H9	-202135.20	-202058.31	-202112.36	70
131	C8N0O0H12	-195837.15	-195761.91	-195807.57	572
132	C2N0O2H6	-144495.54	-144495.54	-144495.54	1
133	C2N5O0H5	-221509.97	-221461.02	-221482.26	10
134	C5N0O3H10	-264984.12	-264916.36	-264950.17	217
135	C3N1O3H3	-248872.82	-248789.90	-248833.84	20
136	C3N1O3H7	-250377.20	-250315.53	-250361.38	5
137	C6N1O1H15	-230726.19	-230700.55	-230712.70	192
138	C6N0O0H6	-145748.37	-145628.06	-145679.31	11
139	C5N1O1H9	-204552.08	-204454.75	-204500.94	449
140	C4N1O3H11	-275776.91	-275770.04	-275774.03	4
141	C3N2O0H2	-141182.95	-141182.95	-141182.95	1
142	C2N4O0H4	-186767.30	-186737.72	-186750.23	8
143	C3N3O1H3	-223159.72	-223069.59	-223123.85	29
144	C4N0O4H2	-285218.63	-285183.15	-285203.99	4
145	C4N2O2H4	-260321.94	-260161.43	-260259.91	411
146	C6N0O2H10	-241692.31	-241591.52	-241643.00	1625
147	C7N0O0H12	-171931.12	-171877.90	-171907.42	122
148	C6N1O0H5	-179699.09	-179607.07	-179661.95	24
149	C1N5O1H1	-243223.85	-243170.71	-243197.28	2
150	C1N1O1H3	-106617.69	-106617.69	-106617.69	1
151	C6N0O1H10	-194474.41	-194399.90	-194437.23	362
152	C8N0O0H6	-193534.29	-193415.62	-193470.31	23
153	C4N1O1H9	-180639.67	-180575.18	-180599.09	67
154	C2N1O1H3	-130501.32	-130501.32	-130501.32	1
155	C3N0O3H4	-214878.67	-214838.90	-214857.36	9
156	C4N2O0H0	-164300.83	-164300.83	-164300.83	1
157	C7N0O0H8	-170422.80	-170304.58	-170355.22	56
158	C2N1O0H7	-84830.48	-84824.75	-84827.62	2
159	C5N1O0H5	-155811.02	-155687.93	-155755.76	10
160	C2N2O0H0	-116506.25	-116506.25	-116506.25	1
161	C3N3O2H5	-271139.44	-271044.64	-271097.03	97
162	C4N1O3H9	-275052.25	-274976.63	-275030.25	49
163	C4N3O1H9	-249362.56	-249293.04	-249331.14	171
164	C1N7O0H1	-264707.43	-264707.43	-264707.43	1
165	C4N0O0H8	-98670.63	-98660.82	-98665.84	5
166	C4N1O3H1	-271984.64	-271936.21	-271958.15	3
167	C3N3O1H7	-224689.15	-224632.89	-224673.41	21
168	C2N0O3H2	-190194.56	-190194.56	-190194.56	1
169	C4N1O1H7	-179877.60	-179792.33	-179836.42	77
170	C5N2O1H4	-236993.52	-236875.44	-236946.72	254
171	C3N5O0H3	-244649.46	-244562.81	-244621.69	23
172	C5N2O0H8	-191316.23	-191198.39	-191271.37	255
173	C5N0O2H2	-214657.03	-214657.03	-214657.03	1
174	C3N2O3H0	-282027.48	-282027.48	-282027.48	1
175	C6N1O1H3	-226135.50	-226053.70	-226100.45	25
176	C4N1O2H3	-225562.52	-225458.32	-225514.01	39
177	C4N2O0H6	-166640.04	-166530.22	-166600.19	45
178	C2N3O1H5	-200014.35	-200004.62	-200008.37	4

179	C1N3O2H1	-221736.72	-221703.55	-221717.69	3
180	C6N1O0H15	-183519.45	-183505.00	-183513.66	38
181	C3N2O2H6	-237167.24	-237119.26	-237142.73	32
182	C4N2O2H2	-259527.03	-259446.23	-259488.93	50
183	C5N3O0H5	-224527.48	-224413.66	-224485.28	233
184	C6N0O1H4	-192138.65	-192073.09	-192102.73	10
185	C2N2O4H0	-305281.64	-305281.64	-305281.64	1
186	C6N0O1H12	-195233.36	-195196.84	-195210.15	192
187	C8N0O0H4	-192694.69	-192676.24	-192685.10	4
188	C5N0O0H12	-124115.81	-124114.51	-124115.05	3
189	C4N1O0H9	-133408.81	-133385.79	-133394.27	19
190	C3N5O0H7	-246181.58	-246133.04	-246156.90	37
191	C5N0O0H8	-122579.32	-122537.30	-122559.00	9
192	C6N1O0H3	-178891.86	-178857.96	-178875.17	4
193	C3N3O1H5	-223927.41	-223855.61	-223892.98	70
194	C5N0O3H6	-263461.79	-263335.69	-263413.26	299
195	C3N4O1H8	-259429.62	-259397.42	-259417.45	13
196	C4N1O2H1	-224753.29	-224753.29	-224753.29	1
197	C4N3O1H7	-248602.24	-248509.15	-248561.48	435
198	C2N6O0H4	-255460.89	-255398.77	-255443.21	13
199	C3N3O0H3	-175940.72	-175898.30	-175917.44	3
200	C3N3O0H11	-178967.62	-178967.62	-178967.62	1
201	C5N1O1H13	-206050.29	-206029.25	-206041.73	61
202	C3N2O1H2	-188377.17	-188377.17	-188377.17	1
203	C0N4O1H0	-184551.81	-184551.81	-184551.81	1
204	C3N0O1H2	-119642.14	-119642.14	-119642.14	1
205	C3N2O1H4	-189182.27	-189125.09	-189155.13	25
206	C1N3O0H5	-128882.75	-128882.75	-128882.75	1
207	C5N1O1H7	-203783.35	-203663.06	-203735.92	349
208	C2N0O0H6	-50099.55	-50099.55	-50099.55	1
209	C4N0O2H2	-190755.00	-190750.86	-190752.93	2
210	C6N1O0H7	-180488.18	-180364.17	-180419.11	87
211	C2N4O1H6	-234762.05	-234762.05	-234762.05	1
212	C3N1O4H5	-296840.85	-296829.30	-296835.08	2
213	C4N0O0H6	-97895.20	-97880.01	-97887.40	3
214	C3N2O0H8	-143483.62	-143449.36	-143469.50	10
215	C5N1O0H3	-154995.96	-154987.79	-154991.92	3
216	C6N0O2H8	-240925.54	-240810.01	-240873.67	1448
217	C1N6O1H0	-277152.20	-277152.20	-277152.20	1
218	C3N2O0H10	-144235.61	-144227.82	-144232.27	3
219	C7N0O0H16	-173458.28	-173454.91	-173456.49	9
220	C6N2O0H12	-216742.17	-216652.86	-216701.92	1486
221	C6N0O0H4	-144899.80	-144888.93	-144893.79	3
222	C2N2O0H6	-118813.32	-118802.82	-118806.49	3
223	C1N4O2H0	-255643.08	-255643.08	-255643.08	1
224	C5N2O1H0	-235404.62	-235404.62	-235404.62	1
225	C3N2O1H0	-187611.88	-187611.88	-187611.88	1
226	C6N0O2H12	-242452.02	-242389.24	-242412.05	691
227	C3N3O1H1	-222350.17	-222288.68	-222330.89	7
228	C3N2O2H2	-235615.76	-235543.27	-235582.51	17
229	C3N4O0H4	-210695.77	-210619.96	-210655.64	15
230	C2N1O3H1	-224152.99	-224152.99	-224152.99	1
231	C4N3O0H3	-199849.22	-199792.49	-199821.39	16
232	C6N0O2H2	-238548.71	-238537.80	-238543.15	4
233	C7N0O0H14	-172701.82	-172675.18	-172682.37	56
234	C5N0O1H8	-169799.95	-169728.54	-169764.17	79
235	C4N1O1H1	-177527.93	-177520.85	-177524.39	2
236	C4N1O2H7	-227089.57	-226990.50	-227043.49	248
237	C3N4O0H2	-209905.52	-209851.00	-209885.47	4
238	C2N4O1H4	-233984.47	-233914.08	-233948.95	20
239	C4N0O0H10	-99443.64	-99443.11	-99443.38	2
240	C3N4O0H6	-211445.26	-211411.74	-211427.42	38
241	C3N2O0H6	-142714.28	-142686.52	-142706.20	6

242	C5N1O2H11	-252525.70	-252438.02	-252475.38	610
243	C2N5O1H3	-267941.89	-267840.51	-267909.00	25
244	C2N1O1H5	-131295.40	-131228.70	-131263.47	5
245	C0N0O1H2	-47958.10	-47958.10	-47958.10	1
246	C5N1O2H5	-250241.03	-250090.72	-250181.35	527
247	C3N0O2H2	-166867.47	-166867.47	-166867.47	1
248	C7N0O1H6	-216865.39	-216724.77	-216783.93	163
249	C2N1O0H5	-84044.02	-84044.02	-84044.02	1
250	C6N2O0H4	-213695.55	-213572.89	-213636.09	42
251	C4N0O3H6	-239552.71	-239462.71	-239520.14	62
252	C2N4O2H2	-280403.57	-280328.12	-280373.28	24
253	C4N1O2H5	-226339.41	-226213.79	-226279.84	185
254	C2N2O2H2	-211703.31	-211649.98	-211674.49	7
255	C6N2O0H8	-215233.24	-215090.01	-215170.33	866
256	C5N1O1H5	-203026.03	-202890.01	-202972.61	116
257	C5N0O1H4	-168249.55	-168181.59	-168215.34	7
258	C5N2O0H2	-188971.91	-188954.86	-188963.38	2
259	C6N0O1H6	-192954.27	-192830.79	-192887.37	82
260	C4N3O1H5	-247843.15	-247736.66	-247797.79	367
261	C3N1O0H1	-106416.23	-106416.23	-106416.23	1
262	C5N1O1H3	-202237.83	-202134.11	-202193.06	21
263	C6N2O0H14	-217499.56	-217451.18	-217471.96	660
264	C2N1O1H7	-132029.55	-132029.55	-132029.55	1
265	C3N1O3H1	-248073.22	-248073.22	-248073.22	1
266	C7N0O1H12	-219147.55	-219071.28	-219109.84	1483
267	C5N1O2H3	-249454.11	-249340.33	-249413.24	72
268	C3N0O1H6	-121217.11	-121184.71	-121195.07	7
269	C4N1O3H7	-274307.30	-274205.38	-274268.05	143
270	C4N0O1H8	-145889.53	-145852.76	-145865.72	22
271	C4N4O0H8	-236118.42	-236070.97	-236098.58	183
272	C2N2O1H2	-164497.34	-164447.43	-164470.57	5
273	C6N2O0H16	-218252.66	-218231.55	-218244.16	112
274	C5N1O0H1	-154209.69	-154209.69	-154209.69	1
275	C4N1O2H11	-228583.51	-228562.01	-228570.63	18
276	C2N0O2H2	-142969.11	-142969.11	-142969.11	1
277	C3N0O4H4	-262096.95	-262075.21	-262086.18	4
278	C7N0O1H14	-219905.42	-219866.80	-219881.88	540
279	C3N3O0H5	-176703.94	-176679.49	-176690.69	21
280	C2N5O1H1	-267142.04	-267097.69	-267116.67	7
281	C2N1O2H3	-177732.99	-177718.17	-177725.50	3
282	C8N0O0H2	-191910.26	-191910.26	-191910.26	1
283	C2N6O0H2	-254672.15	-254658.92	-254665.66	6
284	C4N2O1H8	-214620.97	-214525.60	-214571.15	341
285	C3N1O0H9	-109504.40	-109493.90	-109499.68	4
286	C5N1O0H9	-157326.58	-157256.59	-157288.46	66
287	C4N0O3H10	-241045.27	-241034.59	-241039.96	7
288	C3N0O1H4	-120434.34	-120401.24	-120417.79	2
289	C1N1O0H1	-58627.27	-58627.27	-58627.27	1
290	C2N4O0H6	-187526.49	-187526.45	-187526.47	2
291	C2N1O4H1	-271363.57	-271363.57	-271363.57	1
292	C3N2O1H6	-189946.72	-189864.96	-189918.91	23
293	C4N0O4H4	-285999.37	-285936.43	-285972.92	34
294	C7N0O1H16	-220660.52	-220648.80	-220654.68	72
295	C3N1O2H3	-201643.53	-201598.69	-201622.25	13
296	C1N2O0H4	-94136.73	-94136.73	-94136.73	1
297	C2N3O2H3	-246451.05	-246371.10	-246415.41	17
298	C2N0O1H4	-96537.94	-96510.88	-96524.41	2
299	C6N0O1H8	-193709.19	-193614.99	-193663.86	285
300	C5N2O0H12	-192828.37	-192784.12	-192801.61	171
301	C3N5O0H1	-243837.57	-243818.59	-243828.08	2
302	C3N3O0H7	-177460.72	-177435.02	-177447.28	9
303	C5N1O2H9	-251763.03	-251654.01	-251711.01	1446
304	C7N1O0H11	-205917.60	-205814.80	-205864.49	1389

305	C5N2O1H2	-236203.11	-236145.66	-236180.52	12
306	C5N1O2H7	-251012.21	-250869.50	-250945.71	1482
307	C3N2O2H8	-237910.77	-237884.27	-237900.09	7
308	C3N3O2H7	-271908.35	-271829.24	-271886.54	19
309	C2N2O0H4	-118037.93	-118037.93	-118037.93	1
310	C3N1O3H5	-249634.86	-249552.46	-249608.82	13
311	C5N0O1H6	-169031.65	-168942.45	-168988.56	39
312	C4N4O0H6	-235375.22	-235291.71	-235331.17	116
313	C4N0O1H6	-145113.78	-145056.02	-145091.42	16
314	C5N3O0H11	-226809.47	-226723.45	-226770.84	932
315	C6N1O1H5	-226927.72	-226779.02	-226866.47	296
316	C3N2O0H4	-141962.75	-141917.69	-141942.43	5
317	C7N1O0H13	-206670.00	-206597.79	-206636.84	1366
318	C3N3O1H9	-225441.36	-225416.39	-225424.72	7
319	C6N0O2H4	-239380.51	-239243.36	-239328.83	57
320	C7N0O1H10	-218385.32	-218287.11	-218337.86	1688
321	C5N2O0H10	-192068.36	-191987.37	-192032.83	292
322	C4N3O1H3	-247059.80	-246961.61	-247021.50	97
323	C4N2O2H0	-258723.35	-258723.35	-258723.35	1
324	C8N0O0H14	-196608.08	-196547.98	-196580.24	398
325	C5N0O0H4	-121001.51	-120984.71	-120993.11	2
326	C3N3O2H3	-270379.97	-270249.62	-270330.29	110
327	C2N4O1H0	-232381.70	-232381.70	-232381.70	1
328	C3N2O3H6	-284373.92	-284313.12	-284359.21	9
329	C1N5O0H3	-196811.13	-196786.75	-196798.76	3
330	C2N5O0H3	-220717.12	-220685.26	-220700.03	5
331	C2N5O0H7	-222270.45	-222270.45	-222270.45	1
332	C3N0O3H2	-214104.01	-214070.77	-214087.39	2
333	C4N3O0H5	-200626.28	-200545.27	-200586.90	38
334	C7N0O0H10	-171161.28	-171090.24	-171133.41	126
335	C3N0O3H6	-215634.54	-215590.04	-215618.72	8
336	C7N1O0H9	-205163.36	-205034.78	-205090.31	682
337	C7N0O1H2	-215229.57	-215219.08	-215224.32	2
338	C5N0O0H10	-123351.53	-123332.65	-123338.99	10
339	C4N0O4H10	-288245.14	-288245.03	-288245.09	2
340	C5N2O1H14	-240784.88	-240764.28	-240774.00	83
341	C3N0O4H2	-261320.08	-261303.38	-261310.73	3
342	C6N1O0H9	-181243.87	-181143.65	-181191.27	232
343	C2N0O1H6	-97296.92	-97289.02	-97292.97	2
344	C5N3O0H7	-225303.35	-225163.12	-225249.23	636
345	C4N3O0H13	-203641.62	-203633.04	-203636.80	7
346	C3N4O0H10	-212956.97	-212949.02	-212954.06	3
347	C2N6O0H6	-256192.86	-256192.86	-256192.86	1
348	C5N2O1H8	-238535.16	-238402.40	-238476.92	2141
349	C3N4O1H2	-257122.34	-257049.15	-257080.05	36
350	C4N2O0H12	-168908.19	-168895.47	-168903.60	12
351	C2N0O2H4	-143765.40	-143733.03	-143748.42	3
352	C5N3O0H13	-227567.22	-227517.51	-227537.18	331
353	C2N3O0H7	-153549.96	-153544.98	-153547.54	3
354	C2N3O1H3	-199241.80	-199185.49	-199213.51	13
355	C3N0O2H8	-169172.00	-169163.31	-169166.80	3
356	C3N0O0H4	-73208.38	-73208.38	-73208.38	1
357	C4N2O0H2	-165087.15	-165084.04	-165085.60	2
358	C5N1O0H13	-158848.15	-158836.94	-158843.37	17
359	C5N1O2H1	-248635.77	-248631.76	-248633.35	3
360	C6N1O1H11	-229225.41	-229120.29	-229170.16	2491
361	C5N0O3H12	-265720.44	-265701.14	-265710.72	31
362	C5N2O0H6	-190557.05	-190426.39	-190500.98	98
363	C7N0O1H4	-216037.33	-215955.45	-216005.24	16
364	C4N0O1H4	-144351.69	-144293.37	-144321.02	4
365	C2N3O2H1	-245628.48	-245594.21	-245612.63	3
366	C7N0O0H4	-168796.17	-168758.66	-168777.39	3
367	C2N1O3H3	-224961.40	-224961.40	-224961.40	1

368	C3N2O1H8	-190710.08	-190652.10	-190686.98	20
369	C4N1O0H7	-132654.32	-132590.97	-132618.57	11
370	C5N0O1H2	-167435.10	-167426.06	-167430.58	2
371	C7N0O1H8	-217628.72	-217496.37	-217563.50	742
372	C1N5O2H1	-290390.86	-290388.80	-290389.83	2
373	C6N1O1H1	-225322.34	-225312.13	-225316.12	3
374	C3N0O3H8	-216370.46	-216370.46	-216370.46	1
375	C4N1O0H11	-134177.47	-134165.91	-134172.25	8
376	C3N0O2H4	-167654.75	-167624.63	-167640.04	4
377	C5N0O2H6	-216248.78	-216138.83	-216204.92	174
378	C1N0O1H2	-71856.26	-71856.26	-71856.26	1
379	C4N2O2H8	-261843.51	-261706.85	-261774.00	836
380	C4N2O1H2	-212305.85	-212228.62	-212269.59	19
381	C2N0O0H2	-48528.62	-48528.62	-48528.62	1
382	C5N0O1H12	-171317.16	-171307.31	-171312.17	14
383	C3N1O2H5	-202413.20	-202331.10	-202388.87	21
384	C1N6O0H2	-230730.24	-230730.24	-230730.24	1
385	C2N0O0H4	-49319.38	-49319.38	-49319.38	1
386	C3N1O1H7	-155967.71	-155908.36	-155932.88	17
387	C5N0O1H10	-170561.75	-170524.93	-170538.40	66
388	C1N1O0H5	-60156.94	-60156.94	-60156.94	1
389	C1N4O1H2	-209284.49	-209250.38	-209265.24	4
390	C2N1O2H5	-178504.18	-178487.92	-178496.32	3
391	C3N4O1H6	-258660.99	-258581.76	-258626.84	86
392	C4N2O0H10	-168156.22	-168116.30	-168131.89	46
393	C3N0O0H8	-74771.32	-74771.32	-74771.32	1
394	C2N3O3H1	-292862.40	-292770.90	-292817.30	16
395	C2N1O1H1	-129736.61	-129736.61	-129736.61	1
396	C2N3O0H5	-152781.07	-152781.07	-152781.07	1
397	C6N2O0H10	-215991.65	-215866.67	-215937.70	1509
398	C5N0O2H10	-217781.57	-217715.12	-217741.47	187
399	C2N0O3H4	-190960.68	-190960.68	-190960.68	1
400	C1N2O3H0	-234186.35	-234186.35	-234186.35	1
401	C8N0O0H16	-197374.52	-197344.82	-197353.54	134
402	C3N3O2H1	-269581.17	-269503.60	-269546.09	16
403	C3N0O4H6	-262834.13	-262834.13	-262834.13	1
404	C3N1O1H9	-156705.62	-156692.78	-156701.09	5
405	C4N3O0H11	-202896.04	-202851.86	-202879.44	53
406	C5N0O2H4	-215482.07	-215404.54	-215438.59	33

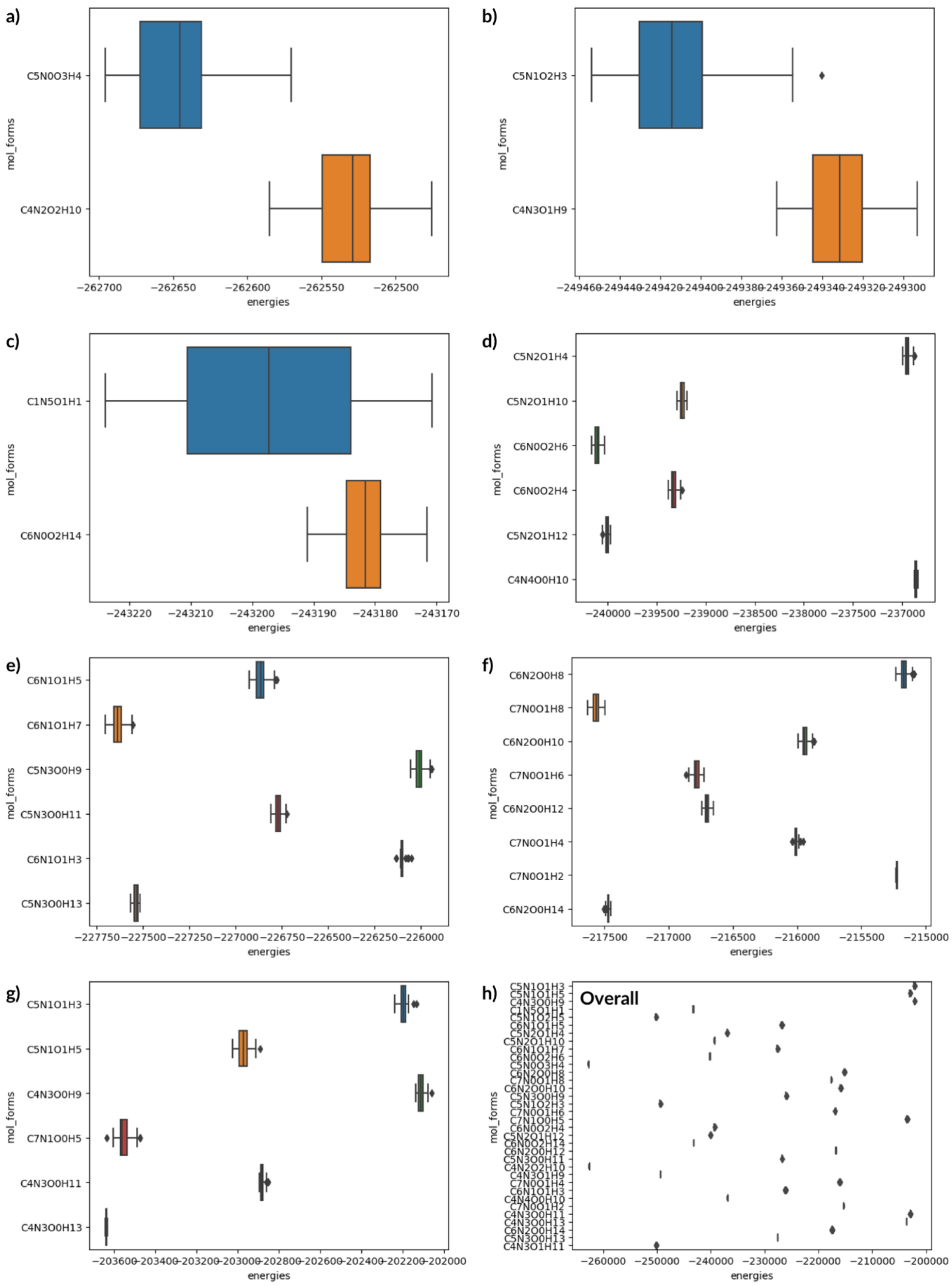

Figure S5. Box-plot diagram of energy distribution of all molecular formulas where the maximum minus the minimum energy range overlaps. The plot g) is the distribution of all the overlapping molecular formulas and the plots a) to g) are expanded, narrower energy range for clarity. The energy unit is kcal mol^{-1}.

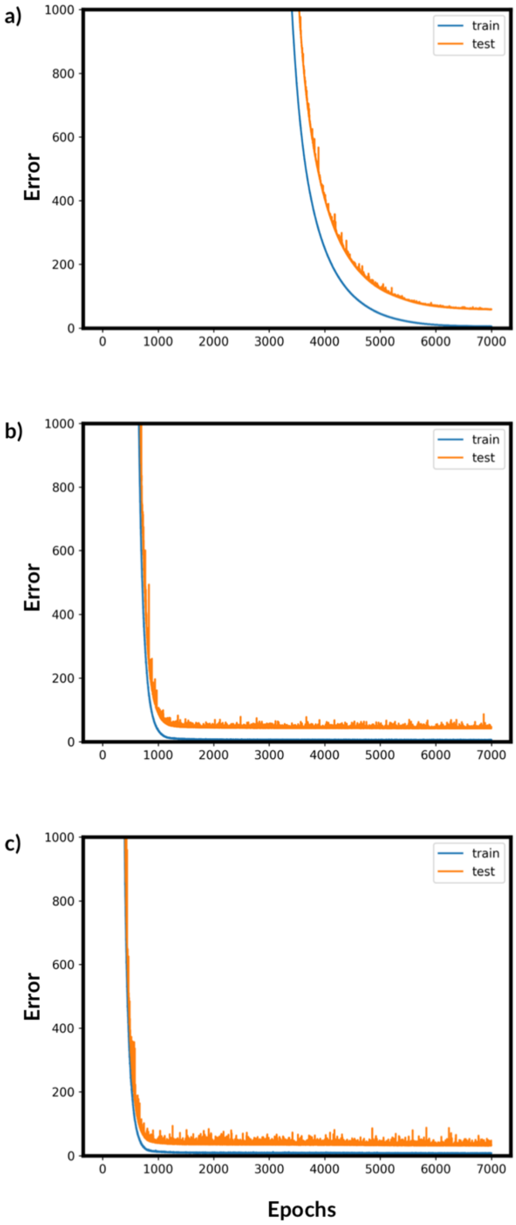

Figure S6. Train and test set error convergence for TrialA2, bond bandwidth 0.07 and angle bandwidth 0.07 neural network training. a) learning rate = 0.0000001, b) learning rate = 0.0000005 c) learning rate = 0.000001

Neural network setup:

All neural networks utilised have two hidden layers, both with the same number of nodes as the input layer (761 features). The two hidden layers have ReLU activation functions and the output layer has the linear activation function. The first hidden layer weights are initialised from the random uniform distribution in the range -500 to 100 and the second and the output weights are initialised from the random uniform distribution in the range 0 to 0.1. The first layer biases are initialised from the random uniform distribution in the range 0 to 10 and the second and the output layer biases are initialised from the random uniform distribution in the range 0 and 0.01. All neural network models are trained using the mean squared error loss function. The weights are trained using the batch size of 64 via the Adam optimiser method.52 The Adam parameters are set to the Keras defaults with β_1= 0.9, β_2 = 0.999 and ϵ = 1.0 × 10-7. The L2 regularisation methods are implemented to avoid overfitting. The layer weight regularisers and bias regularisers are both set to 0.1 in all the hidden layers and the output

layer. Learning rate is an important parameter in ML model training. If the learning rate is too large, the error may diverge during the training and if the learning rate is too small, the training time becomes too lengthy. Optimal learning rate may also be dependent on the number of input features. We therefore train the models with five different learning rates for each representation we test. The training epochs are set to 7000 for all the neural network methods. 7000 epochs are long enough for the training set and the test set error to converge (ESI, Figure S6).

Table S3. Full list of different representations and the learning rates tested. In Trial A1, both the angle and the dihedral connectivity information is included, in Trial A2 only the angle connectivity information is included and in Trial A3 no connectivity information is included. All errors are stated in kcal mol^{-1}.

Representations		Learning Rates					
Trial	Bandwidth	0.00000005	0.0000001	0.0000005	0.000001	0.000005	0.00001
A1	B0.05/A0.05	2.33	1.82	2.68	8.40	45.81	17.97
	B0.05/A0.07	1965.07	2.60	4.71	3.01	582.78	2.43
	B0.07/A0.07	2.70	2.64	1.94	3.67	7.01	3.69
A2	B0.05/A0.05	2.23	2.48	3.59	1.97	20.19	26.08
	B0.05/A0.07	1.95	2.11	2.50	2.42	134.78	11.18
	B0.07/A0.07	31.78	1.80	3.50	2.12	454.52	10.23
A3	B0.05/A0.05	2.85	2.36	2.22	2.45	2.42	59.60
	B0.05/A0.07	2.37	2.20	3.76	2.10	148.25	17.70
	B0.07/A0.07	12.32	17.81	1.94	2.29	766.86	11.46

2.2. Testing different feature generation for NN models

Our initial investigation focused on finding the optimal number of features for our proposed molecular representation. The number of features can be varied by choosing different bandwidths in bond lengths, angles, and dihedral KDE plots. In Figure S7, larger bandwidths have made the KDE plots smoother and the smaller peaks in the CN bond length distribution are not detected as a maximum. Therefore, we explored different bond, angle and dihedral bandwidth combinations as discussed later.

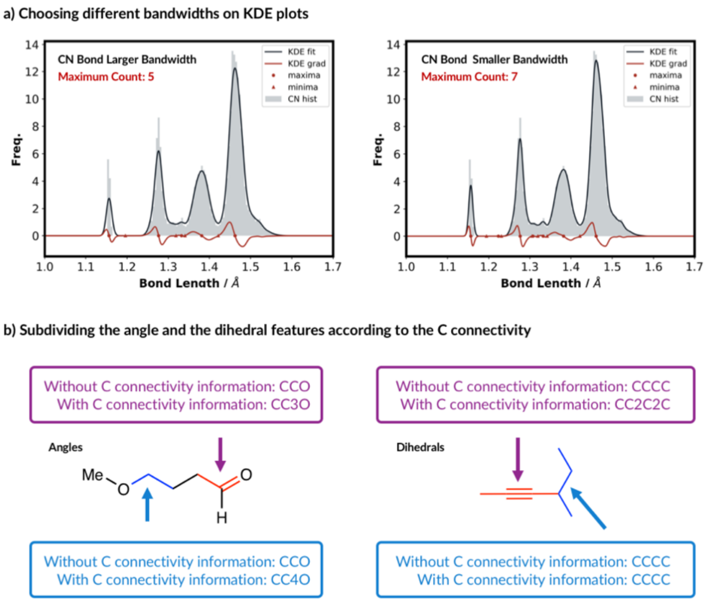

Figure S7. a) Exploring different number of features by changing the bandwidth in KDE plots. The larger bandwidth will make the KDE curves smoother and reduce the number of minima as seen in the left hand side plot. b) Assigning different number of features by recording the connectivity information of the central carbon atoms forming the angles and the dihedrals.

We also varied the number of features by subdividing the angle and the dihedral by the number of connections the carbon atom has. For the angle features, if the central atom within the three atoms forming the angle is carbon, the connectivity information is recorded. The KDE is then plotted separately for the two, three and four connectivity carbons. For example, in the 4-methoxybutanal, CCO angle in the carbonyl and the ether will be assigned to the same CCO group for the KDE plot without the connectivity information but to different groups (CC4O and CC3O) with the connectivity information, Figure S7. For the dihedral features, if the central two atoms within the four atoms forming the dihedral is carbon, the connectivity information is recorded for the 2 connectivity carbons. The KDE is then again plotted separately for the two connectivity dihedrals and the other dihedrals. For example, in 4-methoxy-2-hexyne, the CCCC dihedral across the triple bond and the single bond will be assigned to the same CCCC group for the KDE plot without the connectivity information but to different groups (CC2C2C and CCCC) with the connectivity information.

We have also trained KRR and NN models without the presence of NHx group features and also with the presence of OH group features. For the KRR method, the models trained with NHx, without NHx and with OH gave MAE of 1.232 kcal mol^{-1}, 1.232 kcal mol^{-1} and 1.224 kcal mol^{-1} respectively. For the NN method, the models trained with NHx, without NHx and with OH gave MAE of 1.796 kcal mol^{-1}, 1.802 kcal mol^{-1} and 2.163 kcal mol^{-1} respectively. Therefore, we have chosen the to include NHx group in the feature generation but not the OH groups.

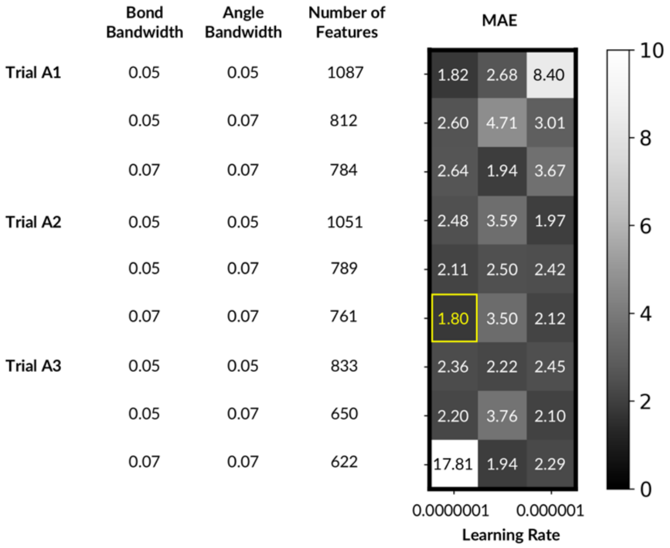

Figure S8. Variety of different representations are tested. In Trial A1, both the angle and the dihedral connectivity information is included, in Trial A2 only the angle connectivity information is included and in Trial A3 no connectivity information is included. The mean absolute errors (MAE) are stated in kcal mol^{-1}. The best performing method is highlighted in yellow.

2.3. ML Models and Hyperparameter Search

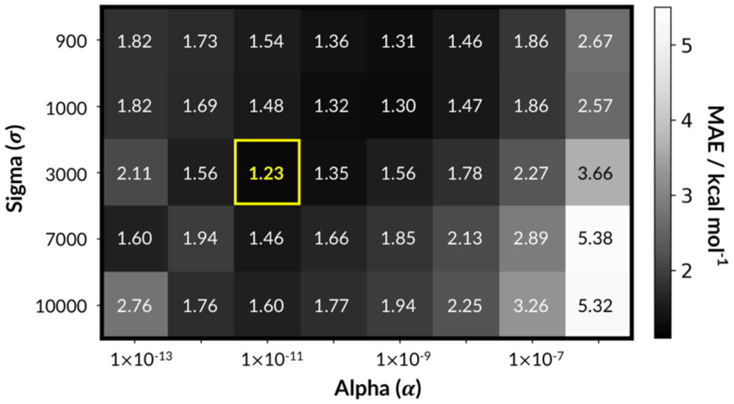

Figure S9. The energy prediction KRR model hyperparameter search. The best performing method is highlighted in yellow

Table S4. The KRR hyperparameter search. The MAEs are stated in kcal mol^{-1}. The best performing method is highlighted in yellow.

sigma	alpha<br>1.00E-13	1.00E-12	1.00E-11	1.00E-10	1.00E-09	1.00E-08	1.00E-07	1.00E-06
100	-	-	-	135.94	135.94	135.95	136.04	137.16
900	1.82	1.73	1.54	1.36	1.31	1.46	1.86	2.67
1000	1.82	1.69	1.48	1.32	1.30	1.47	1.86	2.57
3000	2.11	1.56	1.23	1.35	1.56	1.78	2.27	3.66
7000	1.60	1.94	1.46	1.66	1.85	2.13	2.89	5.38
10000	2.76	1.76	1.60	1.77	1.94	2.25	3.26	5.32

The architectures tested include one hidden layer with q nodes, two hidden layers with q-q nodes, three hidden layers with q-q-q/2 nodes and three hidden layers with q-q-q nodes, where q is the number of features in the input layer. The molecules are allocated to the training and the test set using the molecular formula grouping described in section 2.1. The best performing model has 2 hidden layers, which is the same architecture as section 2.1. Other NN hyperparameters that can potentially affect the performance include the degree of regularisation and the optimiser. We further explored different L2 regularisation factors, ranging from 0.05 to 0.20. We also tested stochastic gradient decent (SGD) and root mean squared propagation (RMSprop) optimisers from Keras for each regularisation, ESI Table S6. The optimal regularisation factor is 0.10. The SGD and RMSprop optimisers do not perform better than the Adam optimiser (section 2.1).

Table S5. NN hyperparameter search: different architectures where q is the number of features in the input layer. The MAEs are stated in kcal mol^{-1}. The best performing method is highlighted in yellow.

Learning Rate	Architecture<br>q	q-q	q-q-q/2	q-q-q	q-q/2	1.5q-q
0.00000001	-	-	err > 1000	err > 1000	-	-
0.00000005	195.16	31.78	err > 1000	err > 1000	39.25	2.21
0.0000001	47.61	1.80	177.96	144.37	2.14	2.22
0.0000005	2.95	3.50	err > 1000	244.74	2.67	4.78
0.000001	2.52	2.12	err > 1000	270.27	-	-
0.000005	2.36	454.52	609.97	53.78	-	-
0.00001	3.73	10.23	err > 1000	err > 1000	-	-

Table S6. NN hyperparameter search: different optimisers and regularisation. The MAEs are stated in kcal mol^{-1}. The best performing method is highlighted in yellow.

Reg	Adam	RMS	SGD
0.05	1.92	45.38	err > 1000
0.1	1.80	32.25	err > 1000
0.15	2.48	4.93	err > 1000
0.2	1.87	7.57	err > 1000

Table S7. MLR hyperparameter search. The MAEs are stated in kcal mol^{-1}. The best performing method is highlighted in yellow.

epochs	alpha				
	0.1	0.01	0.001	0.0001	0.00001
100000	833.43	253.14	33.46	err > 1000	err > 1000
150000	833.31	246.42	25.73	err > 1000	err > 1000
200000	866.22	87.73	26.75	err > 1000	err > 1000

Table S8. The random forest model hyperparameter search: maximum number of samples and maximum number of features. The MAEs are stated in kcal mol^{-1}

max_features	max_samples			
	0.4	0.6	0.8	1.0
761	77.25	53.31	46.28	43.19
700	93.46	64.40	53.55	47.93
600	139.02	101.37	84.53	73.90
500	218.59	168.23	142.05	125.96

Table S9. The random forest model hyperparameter search: maximum tree depth and number of decision trees. The MAEs are stated in kcal mol^{-1}. The best performing method is highlighted in yellow.

N decision trees	max depth = None				max depth = 100				max depth = 20			
	run1	run2	run3	av	run1	run2	run3	av	run1	run2	run3	av
1000	42.69	42.95	42.71	42.78	43.19	42.92	42.74	42.95	43.32	42.88	43.31	43.17
2000	43.12	42.65	42.90	42.89	43.11	42.77	43.15	43.01	42.91	42.84	42.56	42.77
4000	43.01	43.01	43.02	43.01	42.77	42.86	42.78	42.80	43.06	42.88	42.85	42.93
6000	43.13	42.88	42.81	42.94	42.78	42.92	42.86	42.85	42.69	42.64	43.07	42.80

Table S10. Coulomb matrix NN model architecture test. q is the number of features in the input layer. The MAEs are stated in kcal mol^{-1}

Learning Rate	Architecture		
	q	q-q	q-q-q
0.00000001	-	-	err > 1000
0.00000005	759.86	17.01	err > 1000
0.0000001	44.60	64.39	err > 1000
0.0000005	29.78	17.78	84.28
0.000001	29.86	17.13	err > 1000
0.000005	28.89	345.52	174.82
0.00001	29.20	345.51	-

CM, BOB and SLATM NN training:
For the Coulomb matrix representation, the number of input features has been set to 351. NN is set up with the same ReLu activation function for the hidden layers and a linear activation function for the output layer. The weights and the bias are also initialised using the random uniform distribution. We initially tested the one, two and hidden layer NNs with different learning rates, Table S10. The convergence of the training set and the test set errors justified the 7000 epochs used, Figure S10. Further hyperparameter search is then performed on the lowest MAE model, Table S11. Different optimisers and L2 regularisation factors have been tested. Once the optimal hyperparameters have been identified, we trained the NN using the CM representation on the 190, 570, 1900, 5700 molecule training sets. A variety of different learning rates were evaluated as shown in Table S12.

For BOB and SLATM representation, we have set up the NN with ReLu activation function for the hidden layers and a linear activation function for the output layer. The mean squared error was used as the error function with the Adam optimiser. The NN had two hidden layers, each with 351 nodes and 0.1 L2 regularisation. We then trained the NN using the BOB and SLATM representation on the 190, 570, 1900, 5700 molecule training sets. We have again tested a variety of different learning rates for each training set, Table S12. The key results are summarised in Table S13.

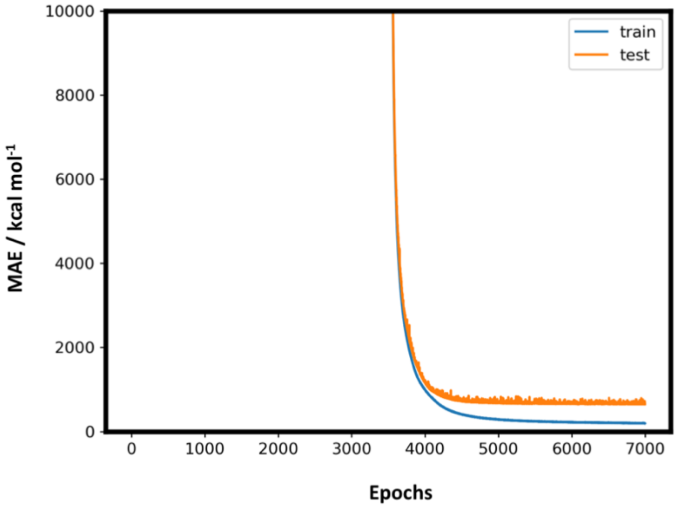

Figure S10. The error convergence for CM representation and NN training. The method used 2 hidden layers, 0.00000005 learning rate, Adam optimiser and 0.10 L_2 regularisation factor

Table S11. CM representation hyperparameter search: optimisers and regularisations

Reg	Adam	RMS	SGD
0.05	16.82	22.71	err > 1000
0.1	17.01	1155.29	err > 1000
0.15	20.83	22.90	err > 1000
0.2	17.34	18.88	err > 1000

Table S12. Performance of CM, BOB and SLATM representations.

N	Lr	MolE8NN	CM	BOB	SLATM
190	10000Lr	err > 10000	2603.32	1439.51	err > 10000
	2000Lr	1143.78	407.34	1024.2	-
	1000Lr	1214.12	422.54	761.58	844.61
	100Lr	3138.20	err > 10000	1469.29	err > 10000
	10Lr	err > 10000	-	226129	226129
570	10000Lr	-	767.15	-	933.17

	1000Lr	195.64	118.07	1121.31	1099.42
	100Lr	94.64	81.74	180.28	593.81
	10Lr	err > 10000	-	226133	err > 10000
1900	10000Lr	-	1972.62	-	222.75
	1000Lr	152.21	52.79	143.96	291.59
	100Lr	402.42	728.57	1548.49	3952.77
	10Lr	13.93	-	36.63	1597.59
	Lr	-	-	26.77	-
5700	10000Lr	-	810.93	-	123.93
	1000Lr	err > 10000	err > 10000	229.81	1306.36
	100Lr	11.81	896.09	991.42	112.22
	10Lr	7.09	28.52	15.41	82.51
full	Lr	1.8	16.82	9.68	16.21

Table S13. Summary of MAE for different training set sizes and ML methods

N	MolE8KRR	MolE8NN	CM	BOB	SLATM
190	96.80	936.08	407.34	761.58	844.61
570	31.62	50.99	81.74	180.28	593.81
1900	5.72	13.93	52.79	26.77	222.75
5700	3.01	7.09	28.52	15.41	112.22
38135	1.23	1.80	16.82	9.68	16.21

2.4 Free energy predictions

Table S14. NN hyperparameter search for free energy predictions. MAEs are stated in kcal mol^{-1}. The best performing method is highlighted in yellow.

Reg	Adam	RMSprop	SGD
0.05	2.53	45.38	err > 1000
0.1	2.56	8.11	err > 1000
0.15	1.92	14.21	err > 1000
0.2	2.65	8.23	err > 1000

Table S15. KRR hyperparameter search for free energy predictions. MAEs are stated in kcal mol^{-1}. The best performing method is highlighted in yellow.

sigma	alpha		
	1.00E-12	1.00E-11	1.00E-10
100	135.24	135.24	135.24
900	1.64	1.46	1.30
1000	1.60	1.40	1.25
3000	1.22	1.16	1.31
7000	1.55	1.42	1.63
10000	2.18	1.57	1.75

2.5. Extrapolation to molecules with 9 or more heavy atoms

KRR Test9 (error > 8 kcal mol^{-1})

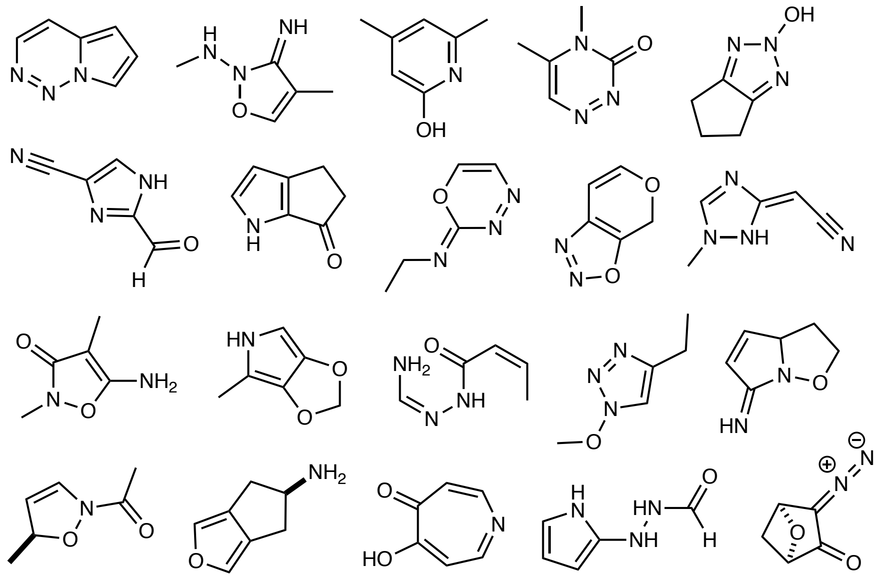

KRR Test10 (error > 13 kcal mol^{-1})

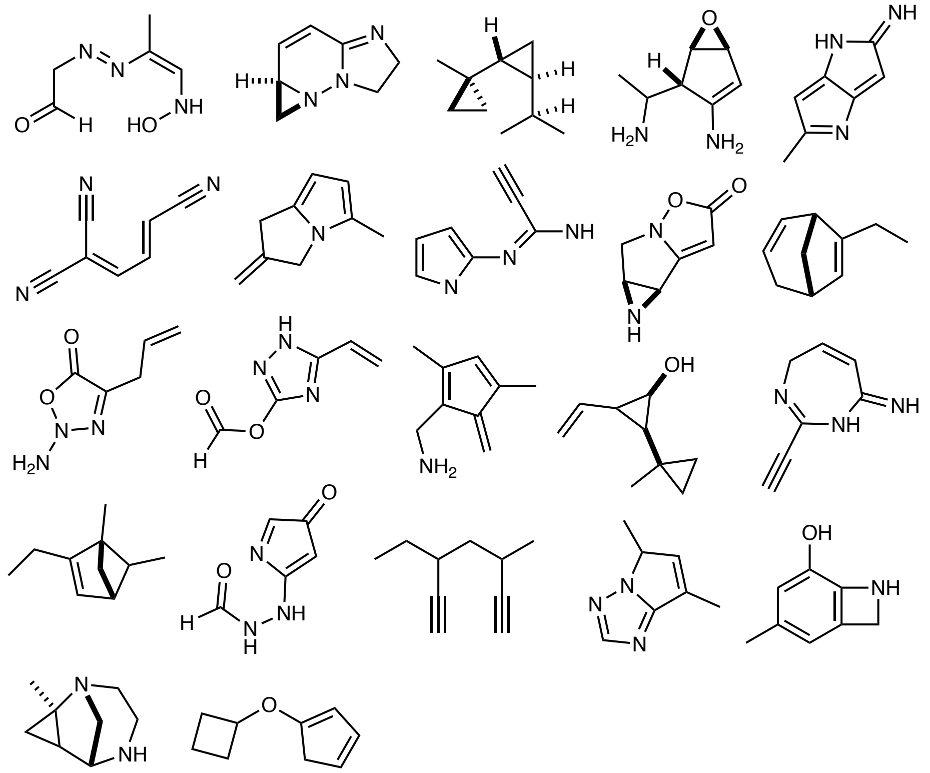

Figure S11. 9 and 10 heavy atom molecules with largest MAE for KRR model

NN Test9 (error > 10 kcal mol^{-1})

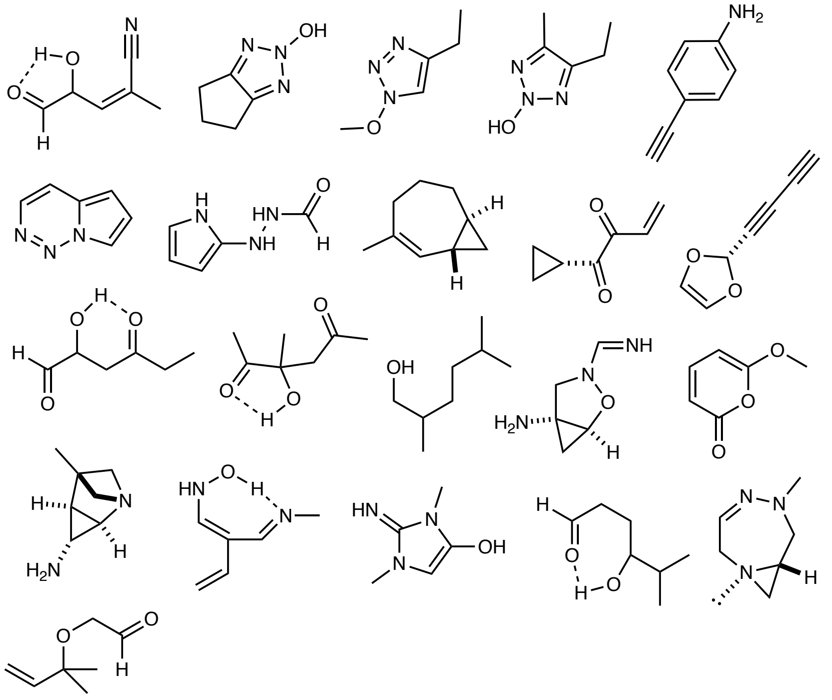

NN Test10 (error > 15 kcal mol^{-1})

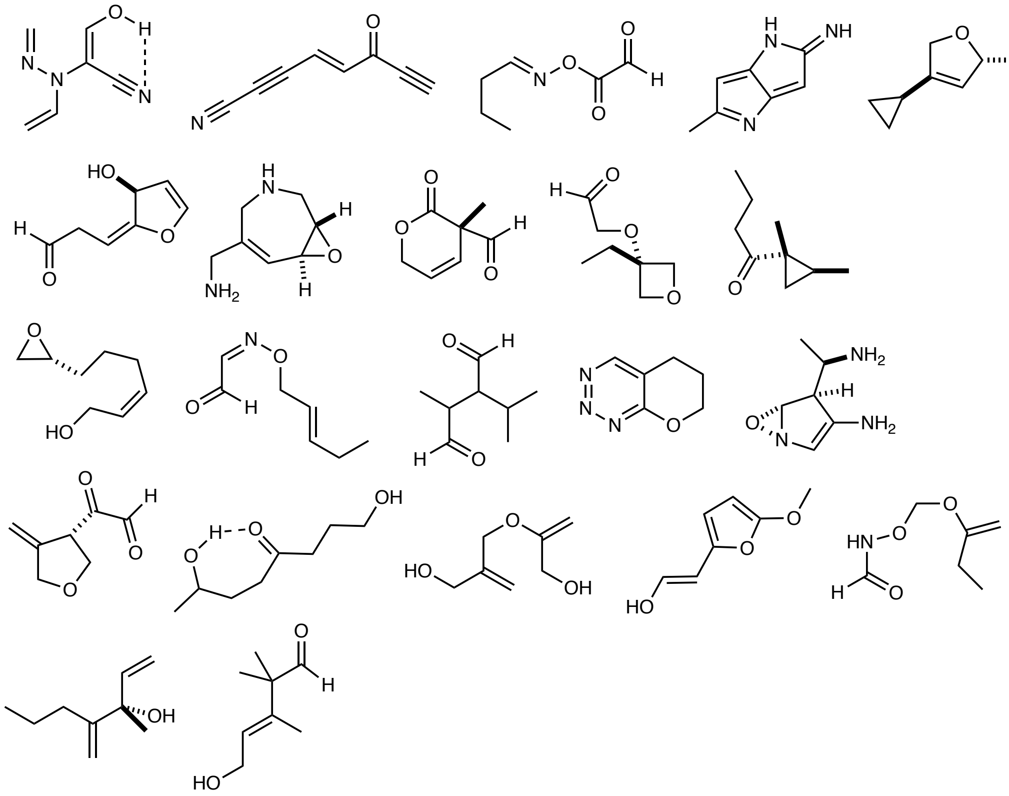

Figure S12. 9 and 10 heavy atom molecules with largest MAE for NN model

2.6. Training with distorted molecules

Table S16. MAEs for NN trained on different number of distorted molecule replicates with 0.01 Å translation for every atom. lr is the learning rate. The MAEs are recorded in kcal mol^{-1} and the predicted properties are DFT energies. The best performing method is highlighted in yellow.

lr / 0p01	Rep1	Rep2	Rep3
0.00000007	1.91	-	-
0.00000005	1.67	1.68	1.67
0.00000002	1.95	1.69	102.85
0.00000001	1.89	42.56	31.13

Table S17. MAEs for NN trained on combination of different distorted molecule replicates. The MAEs are recorded in kcal mol^{-1} and the predicted properties are DFT energies.

Dist. Reps	Dist1	Dist2	Dist3	Test MAE	MMFF MAE
Rep1	0.01	-	-	1.67	4.47
Rep2	0.01	0.05	-	1.70	2.46
Rep2	0.01	0.1	-	1.88	2.46
Rep2	0.01	0.01	-	1.68	3.33
Rep3	0.01	0.01	0.01	1.67	3.30

Table S18. MAEs for KRR trained on combination of different distorted molecule replicates. The MAEs are recorded in kcal mol and the predicted properties are DFT energies. The best performing method is highlighted in yellow.

Dist. Reps	Test Set	MMFF MAE
No Dist	1.23	2.72
Rep1 0.005	1.21	2.54
Rep1 0.01	1.23	2.50
Rep1 0.05	1.46	2.19
Rep1 0.10	1.84	2.45
Rep2 0.01	1.39	2.39
Rep2 0.01 0.05	1.64	2.23
Rep2 0.05 0.10	1.94	2.30
Rep2 0.01 0.10	1.53	2.15

2.7. Extrapolation to 9 or more heavy atoms

Table S19. Energy predictions for 9 or more heavy atom molecules with KRR and NN models trained with and without the distorted molecule replicates

N_atoms	KRR_E	NN_E	KRR_E_Dist	NN_E_Dist
<8	1.23	1.80	1.23	1.70
9	2.06	2.69	2.08	2.15
10	5.18	3.69	5.15	3.01
11	13.87	5.27	13.78	4.21
12	33.46	7.57	33.15	7.11

Table S20. Free energy predictions for 9 or more heavy atom molecules with KRR and NN models trained with and without the distorted molecule replicates

N_atoms	KRR_G	NN_G	KRR_G_Dist	NN_G_Dist
<8	1.18	1.92	1.23	1.87
9	1.89	2.56	2.04	2.22
10	4.70	3.27	5.23	3.15
11	12.32	6.20	13.72	5.37
12	30.94	12.92	33.20	12.86

Table S21. Time taken by MolE8 and TorchANI program to calculate energies of the molecules in the 9-12 heavy atoms database. The time is recorded in seconds for the entire dataset around 1000 molecules. The code was run on 8-core Intel i7 CPU.

	MolE8_KRR/s	MolE8_KRRDist/s	MolE8_NN/s	MolE8_NN_Dist/s	ANI-1
Test9	1.6	3.2	2.2	2.2	10.6
Test10	1.6	3.1	2.2	2.2	11.0
Test11	1.5	3.0	2.2	2.2	11.5
Test12	1.5	3.0	2.3	2.2	13.0

2.8 Further remarks on MLR methods

MLR is the simplest machine learning approach and we can use it to verify that our feature generation method is going in the right direction. In the main text, we have mentioned that MLR gave MAE of 25.7 kcal mol^{-1}. If the predicted energies are completely random, the MAE will be significantly larger than 25.7 kcal mol^{-1}. To justify this point, we have written a code which predicts the energy of the molecules in the test set by generating a random number in between the highest and the lowest value in the y-vector. The MAE then increases to 75360.2 kcal mol^{-1}. We have also performed MLR on coulomb matrix (CM) representation and the resulting MAE is 68.05 kcal mol^{-1}. The results again support our argument that MLR model trained with well-constructed features should perform better than predicting random values.

2.9. Comparison to PhysNet and SchNet

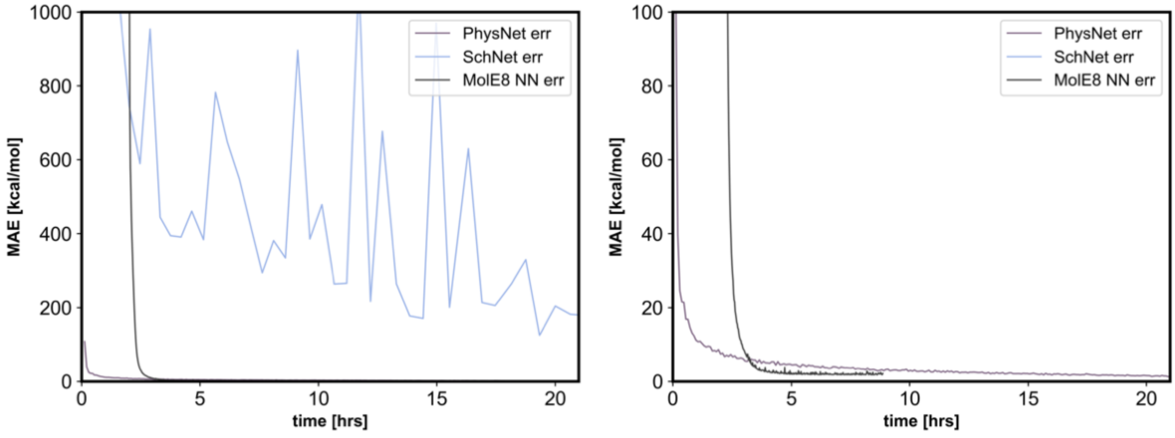

Figure S13. SchNet, PhysNet and MolE8 error convergence comparison

We have used the following configuration file for the PhysNet to generate Figure S13:
--num_features=128
--num_basis=64
--num_blocks=5
--num_residual_atomic=2
--num_residual_interaction=3
--num_residual_output=1
--cutoff=10.0
--use_electrostatic=1
--use_dispersion=1
--grimme_s6=0.5
--grimme_s8=0.2130
--grimme_a1=0.0
--grimme_a2=6.0519
--dataset=CreateFeatures_PhysNet_React8.npz
--num_train=38135
--num_valid=19008
--seed=42
--max_steps=250000
--learning_rate=0.001
--max_norm=1000.0
--ema_decay=0.999
--keep_prob=1.0
--l2lambda=0.0
--nhlambda=0.01
--decay_steps=500000
--decay_rate=0.1
--batch_size=32
--valid_batch_size=1000
--force_weight=52.91772105638412
--charge_weight=14.399645351950548
--dipole_weight=27.211386024367243
--summary_interval=1000
--validation_interval=1000
--save_interval=100
--record_run_metadata=0

We have used the following configuration file for the SchNet to generate Figure S13:


```
{
    "aggregation_mode": "sum",
    "batch_size": 100,
    "checkpoint_interval": 1,
    "contributions": null,
    "cuda": false,
    "cutoff": 10.0,
    "cutoff_function": "cosine",
    "datapath":
"dataset_GDB8/GDB_allgroundnoimagnocarban_Dec2021_G298_LongBondLimit1.6_xyz.db",
    "dataset": "custom",
    "derivative": null,
    "environment_provider": "simple",
    "environment_provider_device": "cpu",
    "features": 128,
    "force": null,
    "interactions": 6,
    "keep_n_checkpoints": 3,
    "log_every_n_epochs": 1,
    "logger": "csv",
    "lr": 0.0001,
    "lr_decay": 0.8,
    "lr_min": 1e-06,
    "lr_patience": 25,
    "max_epochs": 3000,
    "max_steps": null,
    "mode": "train",
    "model": "schnet",
    "modelpath": "model_GDB8_10",
    "n_epochs": 70,
    "negative_dr": false,
    "normalize_filter": false,
    "num_gaussians": 50,
    "output_module": "atomwise",
    "overwrite": false,
    "parallel": false,
    "property": "energy",
    "rho": {},
    "seed": 48,
    "split": [
        38000,
        19000
    ],
    "split_path": null,
    "stress": null
}
```

3. References

46. M. J. Frisch, G. W. Trucks, H. B. Schlegel, G. E. Scuseria, M. A. Robb, J. R. Cheeseman, G. Scalmani, V. Barone, G. A. Petersson, H. Nakatsuji, X. Li, M. Caricato, A. V Marenich, J. Bloino, B. G. Janesko, R. Gomperts, B. Mennucci, H. P. Hratchian, J. V Ortiz, A. F. Izmaylov, J. L. Sonnenberg, D. Williams-Young, F. Ding, F. Lipparini, F. Egidi, J. Goings, B. Peng, A. Petrone, T. Henderson, D. Ranasinghe, V. G. Zakrzewski, J. Gao, N. Rega, G. Zheng, W. Liang, M. Hada, M. Ehara, K. Toyota, R. Fukuda, J. Hasegawa, M. Ishida, T. Nakajima, Y. Honda, O. Kitao, H. Nakai, T. Vreven, K. Throssell, J. A. Montgomery Jr., J. E. Peralta, F. Ogliaro, M. J. Bearpark, J. J. Heyd, E. N. Brothers, K. N. Kudin, V. N. Staroverov, T. A. Keith, R. Kobayashi, J. Normand, K. Raghavachari, A. P. Rendell, J. C. Burant, S. S. Iyengar, J. Tomasi, M. Cossi, J. M. Millam, M. Klene, C. Adamo, R. Cammi, J. W. Ochterski, R. L. Martin, K. Morokuma, O. Farkas, J. B. Foresman and D. J. Fox, 2016, Gaussian, Inc., Wallingford CT, 2016.

48. P. Virtanen, R. Gommers, T. E. Oliphant, M. Haberland, T. Reddy, D. Cournapeau, E. Burovski, P. Peterson, W. Weckesser, J. Bright, S. J. van der Walt, M. Brett, J. Wilson, K. J. Millman, N. Mayorov, A. R. J. Nelson, E. Jones, R. Kern, E. Larson, C. J. Carey, İ. Polat, Y. Feng, E. W. Moore, J. VanderPlas, D. Laxalde, J. Perktold, R. Cimrman, I. Henriksen, E. A. Quintero, C. R. Harris, A. M. Archibald, A. H. Ribeiro, F. Pedregosa, P. van Mulbregt, A. Vijaykumar, A. Pietro Bardelli, A. Rothberg, A. Hilboll, A. Kloeckner, A. Scopatz, A. Lee, A. Rokem, C. N. Woods, C. Fulton, C. Masson, C. Häggström, C. Fitzgerald, D. A. Nicholson, D. R. Hagen, D. V. Pasechnik, E. Olivetti, E. Martin, E. Wieser, F. Silva, F. Lenders, F. Wilhelm, G. Young, G. A. Price, G. L. Ingold, G. E. Allen, G. R. Lee, H. Audren, I. Probst, J. P. Dietrich, J. Silterra, J. T. Webber, J. Slavič, J. Nothman, J. Buchner, J. Kulick, J. L. Schönberger, J. V. de Miranda Cardoso, J. Reimer, J. Harrington, J. L. C. Rodríguez, J. Nunez-Iglesias, J. Kuczynski, K. Tritz, M. Thoma, M. Newville, M. Kümmerer, M. Bolingbroke, M. Tartre, M. Pak, N. J. Smith, N. Nowaczyk, N. Shebanov, O. Pavlyk, P. A. Brodtkorb, P. Lee, R. T. McGibbon, R. Feldbauer, S. Lewis, S. Tygier, S. Sievert, S. Vigna, S. Peterson, S. More, T. Pudlik, T. Oshima, T. J. Pingel, T. P. Robitaille, T. Spura, T. R. Jones, T. Cera, T. Leslie, T. Zito, T. Krauss, U. Upadhyay, Y. O. Halchenko and Y. Vázquez-Baeza, Nat. Methods, 2020, 17, 261–272.
